# CAR T-cells targeting CD117 effectively eliminate mast cells in preclinical models of advanced systemic mastocytosis

**DOI:** 10.1038/s41375-026-02968-5

**Published:** 2026-05-07

**Authors:** Anne Kaiser, Veronika Lysenko, Renier Myburgh, Laura Volta, Christian Pellegrino, Alexandre P. A. Theocharides, Deborah Christen, Jens Panse, Marco M. Bühler, Michel Arock, Joseph Butterfield, Marcelo A. S. de Toledo, Peter Valent, Martin Zenke, Tim H. Brümmendorf, Markus G. Manz

**Affiliations:** 1 Department of Medical Oncology and Hematology, University Hospital Zurich and University of Zurich, Zurich, Switzerland; 2 Department of Hematology, Oncology, Hemostaseology, and Stem Cell Transplantation, Faculty of Medicine, Uniklinik Aachen, RWTH Aachen University, Aachen, Germany; 3 Center for Integrated Oncology Aachen Bonn Cologne Düsseldorf (CIO ABCD), Aachen, Germany; 4 Comprehensive Cancer Center Zurich (CCCZ), Zurich, Switzerland; 5 Department of Pathology and Molecular Pathology, University Hospital Zurich, Zurich, Switzerland; 6 The LOOP Zurich, Zurich, Switzerland; 7 CEREMAST, Department of Hematological Biology, Pitié-Salpêtrière Hospital, Paris Sorbonne University, Paris, France; 8 Divisions of Allergy, Asthma and Immunology, Mayo Clinic, Rochester, Minnesota USA; 9 Department of Internal Medicine I, Division of Hematology and Hemostaseology, Medical University of Vienna, Vienna, Austria; 10 Ludwig Boltzmann Institute for Hematology and Oncology, Medical University of Vienna, Vienna, Austria

**Keywords:** Preclinical research, Translational research, Immunotherapy

## Abstract

Systemic mastocytosis (SM) is characterized by uncontrolled expansion of neoplastic mast cells (MCs) and their accumulation in various tissues and organs, ranging from indolent variants to more advanced forms (advSM). Although several MC- and SM-expressed cell surface antigens have been identified, no immune therapy has been developed for advSM so far. The receptor tyrosine kinase KIT (CD117) is highly expressed on MCs, exceeding the levels of expression on hematopoietic stem and progenitor cells (HSPC). Therefore, targeting CD117 in advSM could be of therapeutic value. In this study, we assessed the therapeutic potential of anti-CD117 chimeric antigen receptor (CAR) T-cells to target neoplastic MCs in SM. In vitro, anti-CD117-CAR T-cells efficiently lysed several SM-related human MC cell lines, MCs differentiated from SM patient-derived induced pluripotent stem (iPS) cells, and neoplastic bone marrow cells obtained from SM patients. Furthermore, in immunocompromised mice engrafted with an advSM-like MC cell line, repetitive applications of anti-CD117-CAR T-cells were able to inhibit MC expansion. These data may pave the way for the development of anti-CD117-CAR T-cell therapies in advSM.

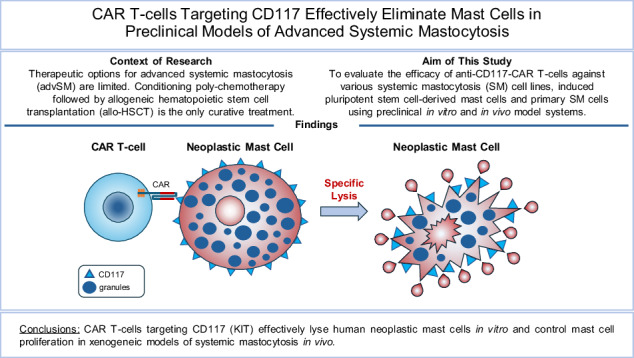

## Introduction

In up to 90% of all patients with systemic mastocytosis (SM), the neoplastic cells harbor the *KIT* D816V mutation. This mutation is located in the intracellular domain of the tyrosine kinase receptor KIT (CD117), leading to constitutive activation of its kinase activity and thereby to ligand-independent differentiation and survival of neoplastic mast cells (MCs) [1–3].

Despite recent advancements in the treatment of SM, such as tyrosine kinase inhibitors (TKIs) like midostaurin [4] and the more specific *KIT* D816V-targeting TKI avapritinib [5–8], advanced forms of SM (advSM: aggressive SM, ASM, SM with an associated hematological neoplasm, SM-AHN, and MC leukemia, MCL [2, 9, 10]) still pose substantial therapeutic challenges. While avapritinib improved the overall response rate to 75% in advSM, including several patients in complete remission, not all patients respond and several relapse after an initial response to avapritinib [5]. In the pre-TKI era, median overall survival ranged from 3.5 years for patients with ASM to less than six months for MCL [11, 12]. Currently, the only available curative approach, especially for TKI refractory patients, is conditioning poly-chemotherapy followed by allogeneic hematopoietic stem cell transplantation (allo-HSCT) [13–17]. This procedure is recommended for young and fit patients as early as possible during the disease course to achieve optimal MC debulking and remission [13–19]. However, allo-HSCT is associated with substantial transplant-related morbidity and mortality as well as a considerable relapse rate, with three-year overall survival of only 43% for patients with ASM and 17% for patients with MCL [16]. These limitations highlight the urgent need for more effective therapeutic strategies.

In pursuit of improved treatment options for advanced and resistant neoplasms in clinical hematology, novel drugs and novel immunotherapeutic strategies, particularly chimeric antigen receptor (CAR) T-cell therapies, have been developed and have shown therapeutic efficacy across various hematological malignancies [20, 21]. The clinical success, which led to the approval of CAR T-cell therapy for B-cell malignancies e.g., [22, 23], paved the way for exploring this approach also for targets expressed on myeloid malignancies [24, 25]. Notably, our group has demonstrated the efficacy of anti-CD117-CAR T-cells and anti-CD117 bispecific T-cell engagers in CD117+ human acute myeloid leukemia (AML) cells both in vitro and in vivo models [26–28]. Given that MCs express CD117 in excess compared to other myeloid cells, we here tested the efficacy of human anti-CD117-CAR T-cells against various SM cell lines and primary SM cells using preclinical in vitro and in vivo model systems.

## Material and methods

### In vitro culture of cell lines and patient-derived cells

Cell lines were established and cultured as previously described [29–32], details are provided in the Supplementary Material and Methods. Isogenic iPS cells with and without *KIT* D816V mutation (Supplementary Fig. 1A) and MC differentiation were previously reported [33, 34]. All patient samples were obtained during routine sampling with informed written consent, in accordance with the Declaration of Helsinki, and were stored at RWTH Aachen University centralized Biomaterial Bank (RWTH cBMB; Project number 41-2020, application number 274, ethical approval number 206/09).

### CAR T-cell generation

Second-generation anti-CD117-CAR T-cells (clone 79D, 4-1BB costimulatory domain) were generated via lentiviral transduction as previously described [26, 27]. A detailed description is provided in Supplementary Material and Methods. Lentiviral particles were produced by transfection of HEK293T cells [27]. The RQR8 gene sequence was kindly provided by Dr. Martin Pule (University College London, UK).

### In vitro studies on CAR T-cell mediated MC lysis

Human MCs (cell lines, iPS cell-derived, bone marrow-derived) were cultured in the appropriate medium (Supplementary Material and Methods). CAR T-cells and control (ctrl.) T-cells were thawed and cultured overnight in T-cell medium. The following day, T-cells were stained with “FarRed dye dilution” to determine T-cell proliferation and co-cultured with target MCs in varying effector-to-target (E:T) ratios in T-cell medium. On the day of analysis, cells were photographed using bright-field microscopy. Cell suspensions were stained with “Zombie Aqua” (BioLegend, San Diego, CA, USA) to assess viability, followed by staining with fluorochrome-conjugated antibodies (Supplementary Table 1). Data acquisition was performed using a “BD LSRFortessa™ II flow cytometer” and “BD FACSDiva™” software (all Becton Dickinson, Franklin Lakes, NJ, USA). Analysis was performed in “FlowJo” (v10.0.7, TreeStar Inc., Becton Dickinson). Specific lysis of target cells was calculated as the ratio of live target cells in co-culture with CAR T-cells to live target cells in the control condition (Supplementary Material and Methods).

### In vivo studies of anti-CD117-CAR T-cell efficacy against the human ROSA^KIT D816V^ MC line engrafting in various mouse models

All animal experiments were approved by the Cantonal Veterinary Office Zurich (license 135/2021) and conducted in accordance with institutional guidelines. Humanized mice (MITRG-SKI and MITRG-SKI-6) were generated and bred in-house as previously described [35–37]. Non-obese diabetic severe common immune deficient gamma (NSG) mice were either in-house bred or purchased from Charles River (Germany) (6–8 weeks old). Detailed information on each in vivo experiment is depicted in the experimental scheme in each figure. In brief, mice were sublethally irradiated with 100 cGy using an “RS-2000 irradiator” (Rad Source, Buford, GA, USA). Four to six hours post-irradiation, human cells were transplanted intravenously (i.v.). For the ROSA^KIT D816V +GFP/Luciferase^ experiments, in vivo bioluminescence imaging was performed to randomize animal groups on day 7 after transplantation and to track cell line engraftment using an “IVIS® Lumina X5 in vivo imaging system” and analyzed with “Living Image” software (both by PerkinElmer, Waltham, MA, USA). Following randomization, 10 ×10^6^ CAR T-cells or control T-cells were i.v. injected 7 and 21 days after cell line transplantation in the treatment experiments. Peripheral blood (PB) was taken by sublingual vein puncture under isoflurane anesthesia for flow cytometry-based monitoring of human engraftment. At terminal analysis, PB, bone marrow (BM), liver, or spleen were examined by flow cytometry analysis or immunohistochemistry (IHC) staining. Histological analyses were conducted according to clinical protocols at the Department of Pathology, University Hospital Zurich, Zurich, Switzerland. For additional information see Supplementary Material and Methods.

### Statistical analysis

All statistical analyses were performed using “GraphPad Prism” (version 10, Boston, MA, USA). Data normality was assessed using the Shapiro–Wilk test. For normally distributed data, comparisons between two groups were made using an unpaired *t* test, while comparisons among more than two groups with unequal variances were analyzed using Brown–Forsythe and Welch one-way ANOVA followed by Dunnett T3 post-hoc tests. For non-normally distributed data, the Mann–Whitney test and Kruskal–Wallis test with Dunn’s multiple comparisons were employed. *P* values were defined as follows: ns, not significant; *p  <  0.05; **p  <  0.01; ***p  <  0.001; ****p  <  0.0001. Error bars represent the mean ± standard deviation.

## Results

### Immunophenotypic characterization of various MC lines

**Fig. 1:**
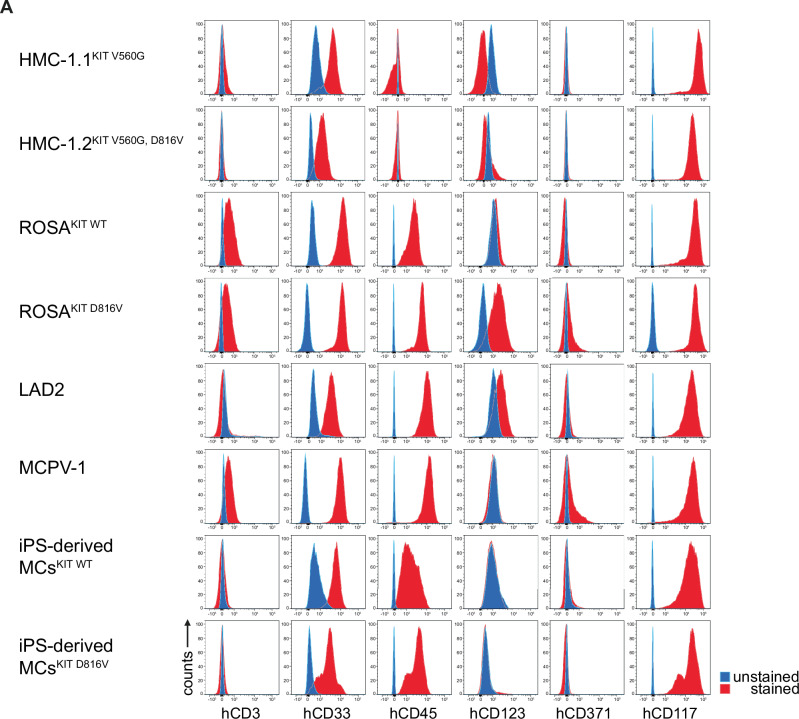
Characterization of various MC populations for target identification. **A** Histograms showing the surface expression patterns of (i) human MC lines (HMC-1.1^KIT V560G^, HMC-1.2^KIT V560G, D816V^, ROSA^KIT WT^, ROSA^KIT D816V^, LAD2, and MCPV-1) and (ii) MCs derived from induced pluripotent stem cells (iPS cells) of a systemic mastocytosis (SM) patient, with or without the oncogenic driver mutation *KIT* D816V as indicated (blue: unstained; red: stained).

We first characterized selected MC lines using flow cytometry (Fig. 1A). In particular, we used HMC-1.1^KIT V560G^, HMC-1.2^KIT V560G, D816V^, ROSA^KIT WT^, ROSA^KIT D816V^, LAD2, and MCPV-1 cells. These cell lines are either derived from patients with SM or generated through genetic modification of cord blood cells and either express or lack the oncogenic driver mutation *KIT* D816V [29–32]. Additionally, we differentiated MCs from SM patient-derived iPS cells, with or without the *KIT* D816V mutation [33, 34]. Patient samples from individuals with MCL, defined by the presence of more than 20% MCs in BM smears [9], were also analyzed by flow cytometry (Supplementary Table 2; Supplementary Fig. 1B). MCs were confirmed by flow cytometry to be CD117^high^/CD45^med^ cells [38]. CD3 was included to distinguish CD3- MCs from CD3+ CAR T-cells. As MCs also express myeloid-associated antigens [38], we included CD33 and found that this target is expressed in all MC samples tested. There was no strong expression of CD123 or CD371 on the given MC types, leading us to discontinue the exploration of targeting these epitopes in our study, unlike in other malignant entities [39, 40]. With the exception of the HMC-1 cell lines, all depicted MC types expressed CD45 and were highly positive for CD117, prompting further investigations to targeting CD117 on MCs by immunotherapeutic means.

### Anti-CD117-CAR T-cells efficiently lyse human CD117-expressing MC lines in vitro independent of *KIT* D816V mutation status

**Fig. 2:**
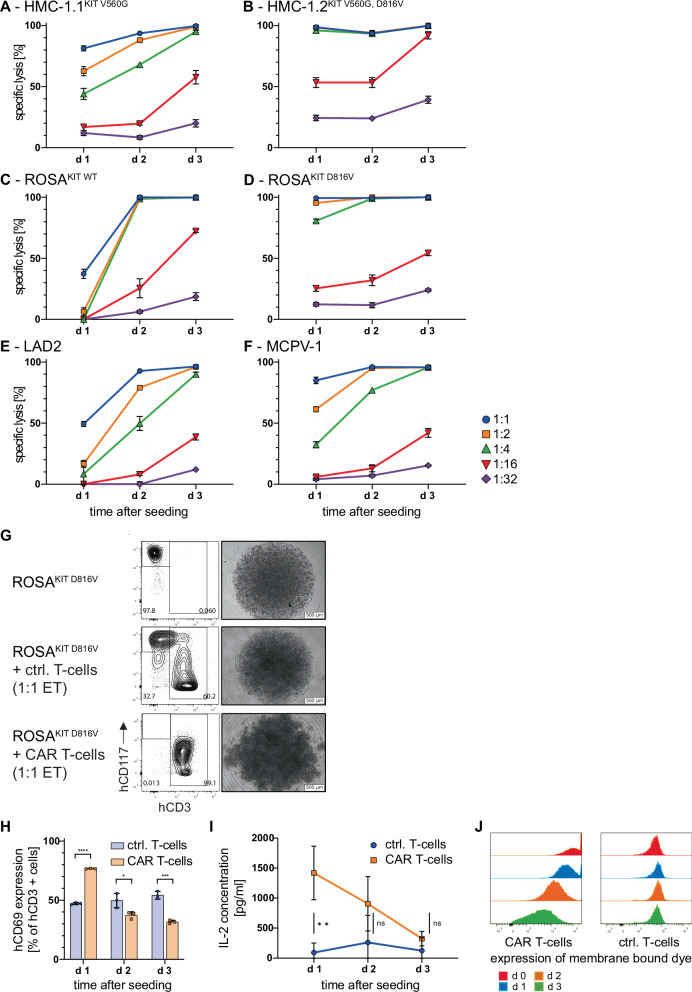
Anti-human CD117-CAR T-cells efficiently lyse human MC lines, regardless of their *KIT* D816V mutation status. **A**–**F** Specific cell lysis of various human MC lines (with or without *KIT* D816V mutation, as indicated) after one, two, or three days of co-culturing with anti-human CD117-CAR T-cells. Lysis percentages were calculated relative to control conditions using untransduced (control: ctrl.) T-cells). Effector-to-target (E:T) ratios ranging from 1:1 to 1:32 are depicted (mean ± SD, blue circle: E:T 1:1, orange square: E:T 1:2, green triangle up: E:T 1:4, red triangle down: E:T 1:16, purple diamond: E:T 1:32). **G** Illustration of ROSA^KIT D816V^ human MC line when cultured alone or co-cultured for three days with anti-human CD117-CAR T-cells (or ctrl. T-cells) at an E:T ratio of 1:1. This is shown through representative flow cytometry plots on the left side, illustrating MC line (hCD117+ cells) alongside T-cells (hCD3+ cells), and on the right side, microscopic views (“Leica DM5500-B” microscope, Leica Microsystems using “Leica LAS-X software” at original magnification x5 with a “HC PL FLUOTAR 5x/0.15” Leica objective; scaling as indicated in lower right corner). **H** Expression of hCD69 on T-cells as a marker for T-cell activation, assessed by flow cytometry (mean ± SD, E:T ratio of 1:1). Statistics: unpaired *t* test: ns; * *p* < 0.05; ** *p* < 0.01; *** *p* < 0.001; **** *p* < 0.0001. **I** IL-2 concentration [in pg/ml] in the co-culture supernatant, measured by ELISA (mean ± SD, E:T ratio of 1:1). Statistics: unpaired *t* test: ns; * *p* < 0.05; ** *p *< 0.01; *** *p* < 0.001; **** *p* < 0.0001. **J** Relative proliferation of T-cells stained prior to co-culturing with “FarRed dye dilution” by assessing the loss of expression of membrane bound dye in the presence of anti-human CD117-CAR T-cells (or ctrl.T-cells) (red: day 0, blue d 1, orange d 2, green d 3, E:T ratio of 1:1). Data shows one example of three independently performed experiments with three healthy donor-derived CAR T-cells, each plated in triplicate wells.

We previously demonstrated the efficacy of anti-CD117-CAR T-cells in lysing CD117+ AML cells [26–28, 41]. Based on the identification of CD117 as a suitable target on the available MC lines (Fig. 1A), we co-cultured CAR T-cells (effector cells = E) and MCs (target cells = T) at decreasing E:T ratios (1:1 to 1:32) for three consecutive days (Fig. 2A–F, Supplementary Fig. 2A–E). Anti-CD117-CAR T-cell activity resulted in over 90% specific lysis of target cells at an E:T ratio of up to 1:4 regardless of the presence of *KIT* D816V mutation. Even at an E:T ratio of 1:32, 10-40% of the target cells were lysed after three days. Figure 2G shows the ROSA^KIT D816V^ cell line cultured alone or co-cultured for three days with control T-cells or anti-human CD117-CAR T-cells at an E:T ratio of 1:1 in representative flow cytometry dot plots. These plots demonstrate effective specific lysis of CD117+ /CD3- MCs by CAR T-cells compared to control conditions. Additionally, CAR T-cell clustering was observed by bright-field microscopy (Fig. 2G). T-cell activation was confirmed by CD69 expression on the CD3+ cell population (Fig. 2H) and IL-2 secretion in cell culture supernatants, measured by enzyme-linked immunosorbent assay (ELISA) (Fig. 2I). Both measures of T-cell activation, CD69 expression and IL-2 secretion, showed significantly higher activation of CAR T-cells compared to control T-cells after one day of co-culture with target cells. CD69 expression was significantly lower in CAR T-cell co-cultures after two and three days compared to control (Fig. 2H). There was no significant difference in CD69 expression of control T-cells throughout the three days (significances not shown). The proinflammatory cytokines tumor necrosis factor α (TNFα) and interferon γ (INFγ) were induced at day 1 and declined at day 2 and day 3 (Supplementary Fig. 3). CAR T-cell activation was accompanied by increased proliferation compared to control (Fig. 2J). Taken together, co-culturing anti-CD117-CAR T-cells in vitro with human MC lines led to effective specific lysis in an E:T- and time-dependent manner, accompanied by phenotypic CAR T-cell activation and proliferation.

### Sustained in vitro efficacy of anti-CD117-CAR T-cells against human MC lines, carrying or not carrying the *KIT* D816V mutation

**Fig. 3:**
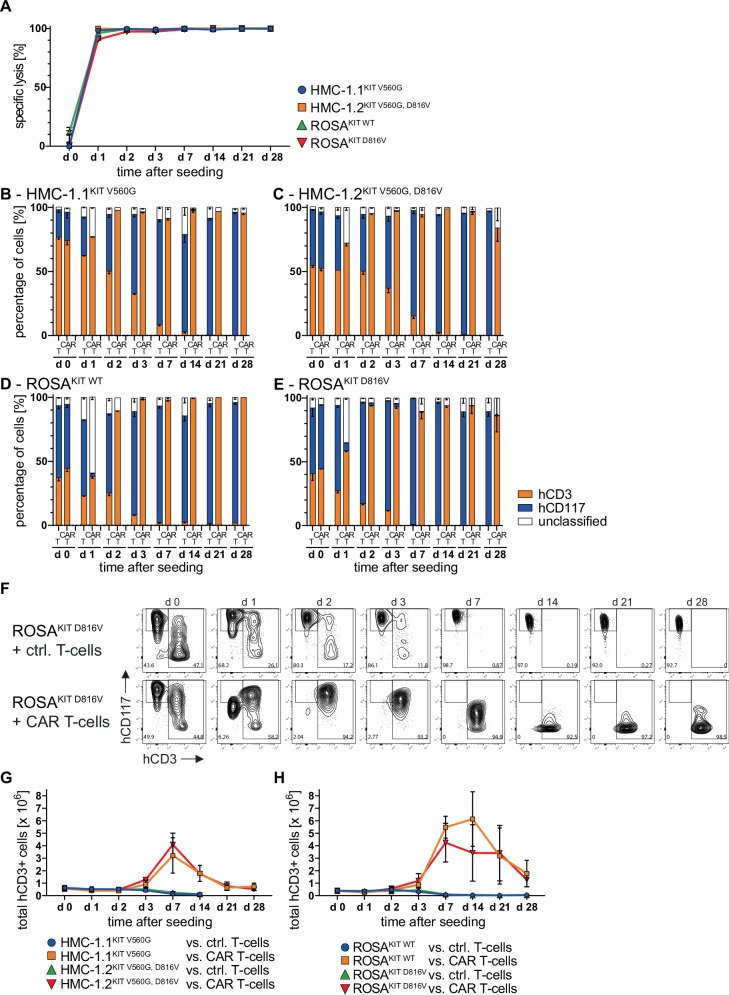
Anti-CD117-CAR T-cells efficiently lyse human MC lines with or without the *KIT* D816V mutation in long-term cultures. **A** Specific cell lysis of different human MC lines (with or without *KIT* D816V mutation as indicated) over a 28-day period of co-culturing with anti-human CD117-CAR T-cells. Lysis percentages were calculated relative to control conditions using untransduced (control) T-cells at an E:T ratio of 1:1 (mean ± SD). **B**–**E** Composition analysis of given cell suspensions shown in percentages (mean ± SD) (T: ctrl.T-cells; CAR T: CAR T-cells). **F** Representative flow cytometry plots illustrating ROSA^KIT D816V^ human MC line (hCD117+ cells) alongside T-cells (hCD3+ cells: either ctrl. T-cells or CAR T-cells) throughout the observative period (d 0 until d 28). (**G**, **H**) Absolute T-cell (hCD3+) counts in given cell suspensions (mean ± SD). Data shows one example of three independently performed experiments with three healthy donor-derived CAR T-cells, each plated in triplicate wells.

To evaluate the sustained efficacy of anti-human CD117-CAR T-cells against human MCs, we co-cultured MC lines expressing or lacking *KIT* D816V (HMC-1.1^KIT V560G^, HMC-1.2^KIT V560G, D816V^, ROSA^KIT WT^, ROSA^KIT D816V^) for 28 days at an 1:1 E:T ratio (Fig. 3) and 1:4 E:T ratio (Supplementary Fig. 4). Specific lysis of target cells by CAR T-cells exceeded 90% after one day of co-culture with all selected human MC lines and remained above 95% throughout the observation period in the 1:1 E:T setting (Fig. 3A) In the 1:4 E:T setting, ROSA^KIT D816V^ MCs were as efficiently controlled as in the 1:1 E:T setting. Interestingly, on day 21 the HMC-1.2^KIT V560G, D816V^ started to outgrow the remaining CD3+ cells. Therefore, this cell line was not efficiently controlled in the reduced E:T ration of 1:4 (Supplementary Fig. 4). In control conditions with T-cells, CD3- MCs expanded in excess over T-cells after seven days, while in the CAR T-cell cultures, more than 95% of the cells were CD3+ CAR T-cells after two days, while 3% of the cells in suspension were CD117+ MCs (Fig. 3B–E). Representative examples of effective lysis of CD117+ /CD3- ROSA^KIT D816V^ MCs by CAR T-cells are shown in Fig. 3F. In the presence of CD117-expressing target cells (MCs), CAR T-cells proliferated, reaching a maximum number of CD3+ cells within seven days, regardless of the *KIT* D816V mutation status of MCs (Fig. 3G, H). In contrast, control T-cell numbers decreased over time as MCs outnumbered these T-cells (Fig. 3B–E). Thus, anti-CD117-CAR T-cells proliferated in the presence of their target antigen and efficiently controlled MC line expansion in vitro throughout a prolonged observation period.

### Anti-CD117-CAR T-cells lyse primary patient-derived MCs and iPS cell-derived MCs in vitro

**Fig. 4:**
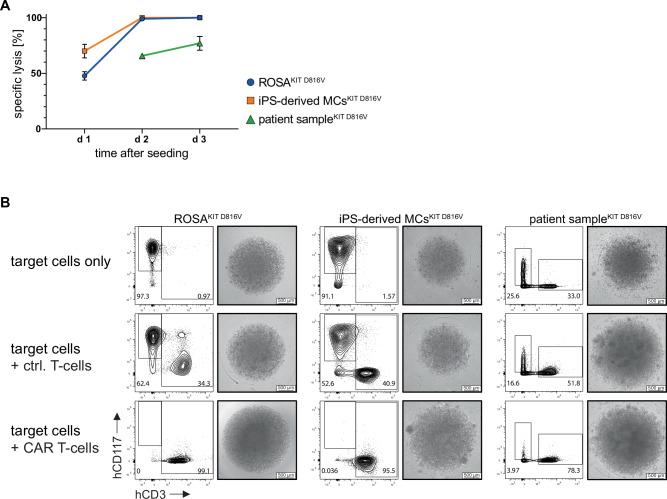
Anti-CD117-CAR T-cells efficiently lyse iPS cell-derived and patient-derived MCs harboring the *KIT* D816V mutation. **A** Specific cell lysis of ROSA^KIT D816V^ human MC line (blue circle), SM patient-derived from iPS cells differentiated MCs (orange square; *KIT* D816V + ), and primary patient sample (bulk bone marrow, green triangle; MCL, *KIT* D816V+ ) after one, two, or three days of co-culturing target cells with anti-human CD117-CAR T-cells (mean ± SD). Lysis percentages were calculated relative to control conditions using untransduced control (ctrl.) T-cells. Effector-to-target (E:T) ratio was 1:1. **B** Illustration of respective human MC material when cultured alone or co-cultured for three days with anti-human CD117-CAR T-cells (or ctrl. T-cells) at an E:T ratio of 1:1. This is shown through representative flow cytometry plots on the left side, illustrating MCs (hCD117+ cells) alongside T-cells (hCD3+ cells), and on the right side, microscopic views (“Leica DM5500-B” microscope, Leica Microsystems using “Leica LAS-X software” at original magnification x5 with a “HC PL FLUOTAR 5x/0.15” Leica objective; scaling bars as indicated in lower right corner). Data shows one example of three independently performed experiments with three healthy donor-derived CAR T-cells, each plated at least in duplicate wells.

We next investigated the in vitro efficacy of anti-CD117-CAR T-cells against MC lines, iPS cell-derived MCs, and primary neoplastic MCs, all harboring the *KIT* D816V mutation (Fig. 4). ROSA^KIT D816V^ cells, *KIT* D816V+ iPS cell-derived MCs, and bulk BM cells (also containing endogenous CD3+ cells, Fig. 4B) of a *KIT* D816V+ advSM patient (patient 2) were co-cultured at an 1:1 E:T ratio either without T-cells, with control T-cells or with anti-CD117-CAR T-cells, each for three consecutive days. After three days, both pure MC populations (ROSA^KIT D816V^ cells and *KIT* D816V+ iPS cell-derived MCs) were effectively lysed by CAR T-cells (Fig. 4A). We also observed the CAR T-cell-induced specific lysis in a vast majority (70%) of CD117+ /CD3- cells in the patient BM sample. Corresponding flow cytometry plots and bright-field microscopic images of the co-cultured cells after three days are displayed in Fig. 4B. These data show that anti-CD117-CAR T-cells effectively eliminate all tested neoplastic MCs exhibiting *KIT* D816V in all MC lines, in iPS cell-derived MCs, and in primary patient-derived cell samples.

### Establishment of a xenograft SM mouse model for evaluation of in vivo CAR T-cell efficacy

Previous studies have established SM murine models relying on the transplantation of MC lines and MCL patient samples into immunocompromised mice [29–31, 42–44]. For example, transplantation of ROSA^KIT D816V^ cells, which resemble advSM [32, 33], into NSG mice caused a MCL-like phenotype with up to 22% MC infiltration in BM after ten weeks [42]. Our previous work showed improved engraftment of human myeloid malignancy cell lines and patient-derived cells in the humanized MISTRG / MITRG-SKI mouse strains (human genes for M-CSF, IL-3, GM-CSF, TPO, and SIRPα knocked into the respective mouse loci) compared to NSG mice [35–37, 45, 46]. Therefore, we sought to investigate whether engraftment of MC material would be further enhanced in the humanized mouse strains MITRG-SKI or MITRG-SKI-6 (which carry an additional *knock-in* of human IL-6 [47], a key cytokine for MC proliferation, maturation and function [33, 34, 48]). For these engraftment experiments healthy and neoplastic MC sources, including CD117+ /CD45+ MCs obtained from a *KIT* D816V + MCL patient (patient 1), ROSA^KIT D816V^ cells, CD34+ progenitor cells from *KIT* D816V+ iPS cells, and CD34+ cord blood-derived healthy donor cells were used.

Transplantation of CD34+ -selected cord blood cells into sublethally irradiated NSG, MITRG-SKI and MITRG-SKI-6 mice showed significant differences in the levels of human CD45+ cells in PB at the time of terminal analysis among the mouse strains (Supplementary Fig. 5C). On the other hand, there was no difference in the percentage between the groups of human CD45 cells in mouse BM at the same time point (Supplementary Fig. 5C). Similarly, the frequency of human CD117+ cells within the human CD45+ population in BM did not differ across mouse strains (Supplementary Fig. 5D). Flow cytometric analysis identified a distinct CD117^high^/CD45^med^ MC population in BM samples, clearly distinguishable from CD117^med^/CD34+ HSPCs (Supplementary Fig. 5E). IHC revealed scattered infiltration of human CD117+ cells in barrier tissues such as skin epithelium, gut mucosa, and spleen (data not shown), consistent with tissue distribution of human MCs [49, 50].

Neither *KIT* D816V+ iPS cell-derived CD34+ progenitor cells ( > 60% CD34+ , Supplementary Fig. 6) nor CD117+ /CD45+ cells from a *KIT* D816V+ SM patient sample (patient 1, Supplementary Fig. 7) engrafted in the humanized mouse strains within a sustainable time frame or amount.

**Fig. 5:**
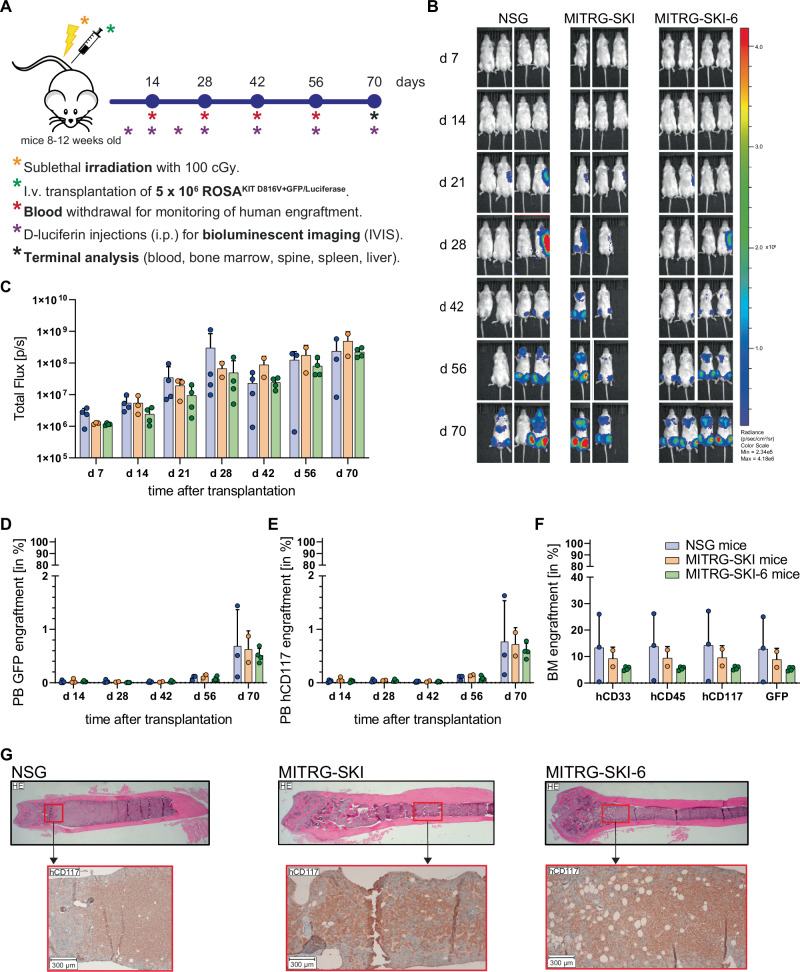
In vivo engraftment of human ROSA^KIT D816V +GFP/Luciferase^ MCs in NSG-, MITRG-SKI- and MITRG-SKI-6 mice. **A** Schematic experimental setup: 5 × 10^6^ ROSA^KIT D816V +GFP/Luciferase^ cells were intravenously (i.v.) transplanted into sublethally irradiated 8–12-week-old NSG (blue, *n* = 4), MITRG-SKI (orange, *n* = 3), or MITRG-SKI-6 (green, *n* = 4) mice. Peripheral blood (PB) was withdrawn every two weeks to assess human engraftment by flow cytometry. As indicated, cell line engraftment was checked by in vivo bioluminescence signal. After 70 days of transplantation, all mice were analyzed. **B** Luminescence imaging. Mouse 1 of NSG- and mouse 3 of MITRG-SKI-group died in anesthesia (engraftment unrelated reason). **C** Total flux of bioluminescence signal [p/s] of ROSA^KIT D816V +GFP/Luciferase^ cells in NSG, MITRG-SKI, or MITRG-SKI-6 mice (mean ± SD). Engraftment of (**D**) GFP+ cells and (E) hCD117+ cells in PB (mean ± SD) over time. **F** Characterization of engrafted human cells in bone marrow (BM) (mean ± SD) on day 70 after cell line transplantation. **G** Representative HE-stained sections of long bones from (i) NSG-, (ii) MITRG-SKI-, and (iii) MITRG-SKI-6 mice, revealing the presence of infiltrated ROSA^KIT D816V +GFP/Luciferase^ cells (brighter areas). Additional immunohistochemical photomicrographs for MCs (hCD117) (scaling bars represent 200 µm).

Subsequently, we proceeded to evaluate the engraftment of ROSA^KIT D816V^ cells, transduced to express green fluorescent protein (GFP) and Luciferase for enhanced in vivo tracking, into NSG, MITRG-SKI, and MITRG-SKI-6 mice (Supplementary Fig. 8B, Supplementary Material and Methods). PB was collected biweekly, and in vivo bioluminescence imaging was used to check for ROSA^KIT D816V+GFP/Luciferase^ cell engraftment (Fig. 5A). After 70 days, the total flux of the bioluminescence signal was comparable across the three mouse strains (Fig. 5B, C). Also, percentages of GFP+ (Fig. 5D) and human CD117+ (Fig. 5E) cells in PB did not significantly differ. Flow cytometric analysis of BM cells showed a slight but not-significant trend towards higher human cell engraftment in NSG mice (Fig. 5F). IHC analysis of long bones confirmed similar BM infiltration by human CD117+ cells in the BM across all mouse strains (Fig. 5G) but only scattered single human CD117+ cells in the liver and the spleen of the NSG mice (Supplementary Fig. 9B and 9C). Flow cytometric analysis did not show significant differences in organ infiltration by CD117+ neoplastic MCs in the liver or the spleen between the given mouse strains (Supplementary Fig. 9B and 9C). Given the comparable engraftment efficiency of ROSA^KIT D816V+GFP/Luciferase^ cells across all three mouse strains, we selected ROSA^KIT D816V+GFP/Luciferase^ in NSG mice as the preferred model to study in vivo efficacy of anti-CD117-CAR T-cells in a xenograft SM mouse model.

### In vivo efficiency of anti-CD117-CAR T-cells against ROSA^KIT D816V +GFP/Luciferase^ human MCs

**Fig. 6:**
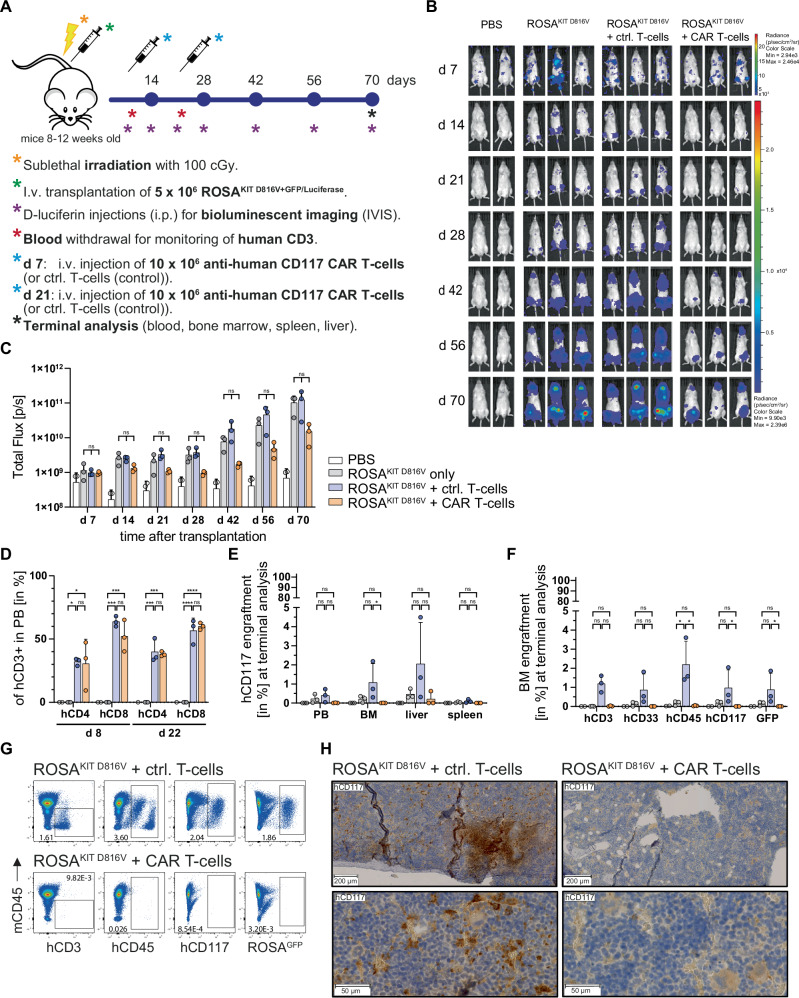
Anti-CD117-CAR T-cells delay in vivo human ROSA^KIT D816V +GFP/Luciferase^ MC growth in NSG mice. **A** Schematic experimental setup: 5 × 10^6^ ROSA^KIT D816V +GFP/Luciferase^ cells (gray, *n* = 3) or PBS (control; white, *n* = 3, one mouse of PBS group died in anesthesia on the day of randomization) were intravenously (i.v.) transplanted in sublethally irradiated 8–12-week-old NSG mice. Randomization of mice based on in vivo bioluminescence signals occurred at day 7 after transplantation. Intravenous injection of 10 × 10^6^ anti-human CD117-CAR T-cells (orange, *n* = 3) or untransduced (ctrl.) T-cells (control; blue, *n* = 3) were administered at day 7 and day 21. Presence of T-cells (hCD3+ cells) in peripheral blood (PB) was assessed at day 8 and day 22 by flow cytometry, while cell line engraftment was monitored throughout the experiment by in vivo bioluminescence signals. After 70 days of transplantation, all mice were analyzed. **B** Luminescence imaging and **C** total flux [p/s] (mean ± SD) of ROSA^KIT D816V +GFP/Luciferase^ cells in NSG mice after respective treatments. **D** Presence of T-cells in PB at day 8 and day 22 after transplantation and subcharacterization of respective hCD4+ and hCD8+ T-cells (mean ± SD). **E** Percentage of MCs (hCD117+ cells) in PB, BM, liver, and spleen (mean ± SD). **F** Percentages of human engraftment in BM by marker expression (mean ± SD). **G** Characterization of engrafted human cells in BM (mean ± SD). **H** Representative IHC analysis for MCs in long bones of mice treated with ctrl.T cells (left) and anti-human CD117-CAR T-cells (right) (scaling bars as indicated in lower left corner). For all statistics: Brown–Forsythe and Welch one-way ANOVA followed by Dunnett T3 post-hoc tests; for non-normally distributed data: Mann–Whitney test and Kruskal–Wallis test with Dunn’s multiple comparisons: ns; * *p* < 0.05; ** *p *< 0.01; *** *p* < 0.001; **** p < 0.0001.

Finally, we assessed the efficacy of anti-CD117-CAR T-cells against ROSA^KIT D816V+GFP/Luciferase^ cells in vivo. We transplanted ROSA^KIT D816V+GFP/Luciferase^ cells into NSG mice (as described above and Supplementary Material and Methods), and on days 7 and 21 after ROSA cell transplantation administered either 10 × 10^6^ anti-human CD117-CAR T-cells or 10 ×10^6^ control T-cells into these mice i.v. (Fig. 6A). The presence of ROSA^KIT D816V+GFP/Luciferase^ cells was tracked by bioluminescence imaging. In both untreated and control T-cell group, the bioluminescence emission increased continuously, suggesting ROSA^KIT D816V+GFP/Luciferase^ cell engraftment and expansion. In contrast, the bioluminescence emission initially remained stable and then showed a less pronounced increase in the CAR T-cell-treated group compared to the control groups (Fig. 6B, C). The presence of human CD3+ cells, predominantly CD8+ cells, was detected in the PB one day after each CAR T-cell or control T-cell injection (Fig. 6D). At terminal analysis, no cells expressing GFP or human markers, particularly human CD117, were detectable in any investigated organ (PB, BM, liver, and spleen) in the CAR T-cell treatment group by flow cytometry (Fig. 6E, F). A representative flow cytometry plot of BM illustrates complete elimination of ROSA^KIT D816V+GFP/Luciferase^ cells in CAR T-cell-treated animals, while cells remained detectable in control T-cell-treated animals (Fig. 6G). Interestingly, CAR T-cells were no longer detectable upon ROSA cell elimination, whereas control T-cells remained present. Furthermore, IHC staining of the long bones confirmed complete elimination of human CD117+ infiltration in the CAR T-cell cohort while CD117+ cells were clearly observed in the control group (Fig. 6H). IHC staining of the liver and spleen neither revealed human CD117+ infiltration nor architectural differences of the tissues after CAR T-cell treatment compared to control (Supplementary Fig. 10). We thus hypothesize that the emitted bioluminescent signal must have been derived from some extramedullary engraftment which could not be controlled by the anti-CD117-CAR T-cells.

Although not statistically significant, the comparison of the control groups (ROSA^KIT D816V+GFP/Luciferase^ cells vs. ROSA^KIT D816V+GFP/Luciferase^ cells and T-cells) might suggest that ROSA^KIT D816V+GFP/Luciferase^ cells engraft slightly better in presence of T-cells. If indeed true, this might be explained by T-cell produced factors, e.g. IL-3, which could foster MC growth.

Together, the here presented data demonstrate that anti-CD117-CAR T-cells can control the growth of CD117-expressing MCs in vivo in a xenogeneic mouse model system.

## Discussion

CAR T-cell therapies are effective in B-cell and plasma cell malignancies [20, 21]. Although less advanced, they also hold promise in myeloid neoplasms and advSM. Previously, only a few immunotherapeutic approaches have been investigated in SM contexts, but no CAR T-cell therapies have been established. Here we describe the effectivity of anti-CD117-CAR T-cells in targeting neoplastic MCs in preclinical advSM models.

Omalizumab, a humanized anti-IgE neutralizing antibody, is effective in allergic diatheses and skin symptoms associated with ISM [51]. However, as expected, omalizumab yielded low efficacy in reducing MC burden in these patients. Similarly, gemtuzumab ozogamicin (GO), which targets CD33-expressing MCs delivering a cytotoxic payload, has shown promising in vitro data [43, 52], but only a few effective applications in patients were reported thus far [13, 43, 53]. The identification of CD52 expression on MCs led to the use of alemtuzumab, which prolonged survival in a xenograft mouse model and reduced the MCL-like phenotype [29]. Additionally, in vitro studies showed promising results of brentuximab-vedotin targeting CD30+ MC lines and primary neoplastic MCs [44] but failed to demonstrate efficacy in a phase 2 clinical trial involving advSM patients [54], probably attributed to the fact that neoplastic stem cells in advSM do not express CD30 [43]. So far, KIT kinase activity has been explored as a target of TKI in SM contexts, and indeed these are potent disease-modifying agents in advSM patients [4–8].

In line with previous work, characterizing healthy and neoplastic MCs as CD117^high^/CD45^med^ [38], our data confirmed high expression of CD117 across human MCs, including cell lines, iPS-cell derived MCs, and primary SM patient samples. The strong, broad and homogenous distribution of CD117 on all MC types provides an appealing basis for developing an anti-CD117 targeted CAR T-cell approach. However, CD117 is not exclusively expressed on MCs but also on other cell types in various tissues [27, 55] raising concerns about potential on-target off-tumor effects of anti-CD117 directed therapies lysing CD117+ cells independent of their CD117 expression level. One major concern is on-target lysis of HSPCs expressing CD117. Therefore, anti-CD117-CAR T-cell therapy in eligible advSM patients, especially those with AHNs e.g. AML with high-risk somatic mutations [13–19], might become a valuable option for conditioning therapy prior to alloHSCT, targeting both advSM cells and HSPCs [13–19, 56–58]. Furthermore, while the CD117-targeting antibody briquilimab is efficient as conditioning regimen for alloHSCT eliminating healthy CD117+ HSPCs [58], it might not be sufficient to eliminate aggressive malignant cells as occurring in advSM. Therefore, our proposal on employing CD117-directed CAR T-cells builds on the increasing efficacy to inducing cell death from antibodies to antibody drug conjugates, to bispecific T-cell engaging antibodies (TCE), to second-generation CAR T-cells.

Clearly, anti-CD117-CAR T-cells would need a safety switch for their elimination prior to an incoming alloHSPC transplant. This could be achieved by the application of rituximab to target the CD20 epitope of RQR8, by anti-thymocyte globulin (ATG), or by inducible caspase 9 [26, 27]. Promising approaches to enhance control in anti-CD117 immunotherapy are a “universal” CAR T-cell approach, linking the effector CAR T-cells to the target cell by an adaptor, or a TCE linking endogenous T-cells to the tumor cell, as shown before by others and us [26–28, 41].

Furthermore, there might be a therapeutic window between targeting highly CD117-expressing MCs and lower CD117-expressing HSPCs [28, 38], which should lead to preferential MC killing over HSPC killing. Due to experimental limitations, we could not exploit the higher CD117 expression on MCs versus lower expression of CD117 on HSPCs (as seen in Supplementary Fig. 5E) and their cell lysis by defining a therapeutic window in this study. In line with this notion, in a recent study Thomas et al. observed a dependency of effective CAR T-cell mediated CD117+ murine target cell lysis by epitope concentration on the surface [59]. However, so far xenogeneic models for further studies which harbor engrafted human HSPCs, MCs and SM cells are currently not established.

Minimizing further toxicity and organ function impairment by CAR T-cell conditioning should be considered, especially for advSM patients with already pre-existing organ damage. Neoplastic stem cells in SM exhibit considerable amounts of KIT [43] and thus anti-CD117-CAR T-cell therapies may also introduce disease-modifying or even curative effects in SM patients. From our perspective, anti-CD117-CAR T-cell therapy should primarily be considered for advSM patients. Patients with advSM have a reduced life expectancy and, depending on the mutational load, a poor prognostic outcome (e.g. patients with mutations in ASXL1/SRSF2/RUNX1) [9, 11]. These patients are expected to benefit from anti-CD117-CAR T-cell therapy prior to alloHSCT as debulking of CD117+ MCs and CD117+ hematopoietic stem cells might reduce the currently high relapse rates after alloHSCT. In these cases, clinical trials are required to investigate whether the combination of CAR T-cells with or without already established therapies (e.g. TKIs) and conditioning regimes are safe and effective. Additionally, *KIT* D816V negative advSM patients or patients with aspects that prevent TKI-based therapies, like severe thrombocytopenia, might benefit from CAR T-cell therapy as it targets all CD117+ cells independent of their mutational status. For ISM, being not associated with a reduced life expectancy and organ dysfunction [9, 11], alloHSCT is medically not justified and therefore presently anti-CD117-CAR T-cells appears not to be an option.

A potential risk is the release of MC mediators with potentially life-threatening anaphylaxis during CAR T-cell therapy due to rapid MC lysis. In addition, CAR T-cell therapy might be associated with a cytokine release syndrome, which can be aggravated by additional MC mediator release. However, MC lysis induced by CAR T-cells might also be followed by rapid removal of such lysed MCs by macrophages and histiocytic cells. Additionally, no allergic reactions were observed when CAR T-cells were applied to CD117+ AML- [26, 27] or the MCL-model engrafted mice employed in this study.

In summary, our study demonstrates as a proof-of-concept the effectivity of CAR T-cells directed against *KIT* D816V+ MCs in preclinical models of advSM. This anti-CD117-CAR T-cell approach may also provide a more specific conditioning tool prior to allo-HSCT in patients with insufficient therapeutic alternatives.

## Supplementary information

Supplementary

## Data Availability

The datasets generated during and/or analysed during the current study are available from the corresponding author on reasonable request. Markus G. Manz, Department of Medical Oncology and Hematology, University Hospital Zurich and University of Zurich, Zurich, Switzerland, Rämistrasse 100, 8091 Zurich, Switzerland, e mail: markus.manz@usz.ch.
