# Supplementary material for: CAR T-cells targeting CD117 effectively eliminate mast cells in preclinical models of advanced systemic mastocytosis
Source: Leukemia. 2026 May 7;40(7):1482–93. doi: 10.1038/s41375-026-02968-5 (PMC13323065; doi:10.1038/s41375-026-02968-5)
Supplement: Supplementary file 1 — Supplementary [file 41375_2026_2968_MOESM1_ESM.docx]

Supplementary Materials and Methods, Tables & Figures

**CAR T-cells Targeting CD117 Effectively Eliminate Mast Cells in Preclinical Models of Advanced Systemic Mastocytosis**

Anne Kaiser^1,2,3^, Veronika Lysenko^1^, Renier Myburgh^1^, Laura Volta^1^, Christian Pellegrino^1^, Alexandre P.A. Theocharides^1,4^, Deborah Christen^2,3^, Jens Panse^2,3^, Marco M. Bühler^4-6^, Michel Arock^7^, Joseph Butterfield^8^, Marcelo A.S. de Toledo^2,3^, Peter Valent^9,10^, Martin Zenke^2,3^, Tim H. Brümmendorf^2,3^, and Markus G. Manz^1,4,#^.

Affiliations:

^1^Department of Medical Oncology and Hematology, University Hospital Zurich and University of Zurich, Zurich, Switzerland.

^2^Department of Hematology, Oncology, Hemostaseology, and Stem Cell Transplantation, Faculty of Medicine, Uniklinik Aachen, RWTH Aachen University, Aachen, Germany.

^3^Center for Integrated Oncology Aachen Bonn Cologne Düsseldorf (CIO ABCD), Aachen, Germany.

^4^Comprehensive Cancer Center Zurich (CCCZ), Zurich, Switzerland.

^5^Department of Pathology and Molecular Pathology, University Hospital Zurich, Zurich, Switzerland.

^6^The LOOP Zurich, Zurich, Switzerland.

^7^CEREMAST, Department of Hematological Biology, Pitié-Salpêtrière Hospital, Paris Sorbonne University, Paris, France.

^8^Divisions of Allergy, Asthma and Immunology, Mayo Clinic, Rochester, Minnesota, USA.

^9^Department of Internal Medicine I, Division of Hematology and Hemostaseology, Medical University of Vienna, Vienna, Austria.

^10^Ludwig Boltzmann Institute for Hematology and Oncology, Medical University of Vienna, Vienna, Austria.

^#^Corresponding author: Markus G. Manz, University Hospital Zurich and University of Zurich, Zurich, Switzerland, Rämistrasse 100, 8091 Zurich, Switzerland, phone: +41 44 255 38 99, e mail: markus.manz@usz.ch. For original data, please contact the corresponding author Markus G. Manz.

# Methods

# Mast cell material

All cells were cultured under sterile conditions in humidified air with 5% CO2 at 37°C. Mast cell (MC) lines were cultured as previously described^1–4^, mainly in IMDM + L-glutamine medium supplemented with 10% fetal bovine serum (FBS) and 5000 µg/U/ml penicillin/streptomycin (100 U/ml/100 µg/ml; Gibco™ Life Technologies (Carlsbad, CA, USA), Thermo Fisher Scientific (Waltham, MA, USA) with or without 80 – 100 ng/ml human stem cell factor (SCF; Miltenyi Biotec, Bergisch Gladbach, Germany). For further information, see Supplementary Table 3.

## Differentiation from patient-derived iPS cells to mature MCs

Culture of patient-derived iPS cells (corresponding iPS cells derived from patient 3 (nomenclature of Toledo et al. 2021)) and differentiation to mature MCs was performed as previously described^5–8^. In brief, iPS cells were cultured on Matrigel-coated (Corning (Corning, NY, USA), after manufacturer’s instructions) 6-well plates in “StemMACS iPS-Brew XF” (Miltenyi Biotec). For passaging of the iPS cells 0.2 nM EDTA (Thermo Fisher Scientific) PBS (Gibco™ Life Technologies) solution was used. For passaging the iPS cells to initiate a differentiation at a confluency of 60-70% “Accutase™“ (Stemcell Technologies, Vancuver, Canada) was used. On day 0 of differentiation, 5 x 10^5^ iPS cells per of a 96-round bottom well suspension plate (Greiner, Kremsmünster, Österreich) were seeded in the corresponding day 0 medium (Supplementary Table 4) and centrifuged at 392 x g for 5 minutes (leading to formation of spinned embryonic bodies (spinEBs). On day 2, the dedicated medium (Supplementary Table 4) was added. From day 3 to day 13, 50 µl of supernatant medium was carefully removed as to not disturb the forming EB, and 50 µl of new medium of the corresponding medium was added. On day 14, EBs and hematopoietic stem- and progenitor cells (HSPCs) were harvested, centrifuged and resuspended in fresh progenitor medium (Supplementary Table 4). Progenitor medium was exchanged while keeping the cell density between 1-2 x 10^6^ cells/ml. After 14 to 21 days, progenitor cells were either harvested and injected in mice for engraftment experiments or subjected to fluorescent activated cell sorting (BD FACSAria cell sorter, Becton Dickinson, Franklin Lakes, NJ, USA) to gain a hCD117+/hCD45+ population (Supplementary Table 1). Further differentiation to mature MCs took up to 70 days of cell culture.

## Patient samples

All bone marrow bulk cell samples from patients with a confirmed diagnosis of advSM were acquired in context of routine sampling upon informed and written consent, in accordance with the Declaration of Helsinki, and stored at RWTH centralized Biomaterial Bank (RWTH cBMB) (Project number 41-2020, application number 274, ethical approval number 206/09). After withdrawal from the Biomaterial Bank patient samples were thawed in 50 ml 1:1 IMDM (Gibco™ Life Technologies): FCS (Gibco™ Life Technologies), 0.2 mg/ml DNAse (Roche/Sigma-Aldrich, St. Louis, MO, USA), 5 ng/ml SCF (Miltenyi Biotec), and 2 mM EDTA (Thermo Fisher Scientific) and incubated for 30 minutes at 5% CO2 and 37°C. Afterwards cells were centrifuged and resuspended in T-cell medium (advanced RPMI media supplemented with 10% FBS, penicillin/streptomycin (100 U/ml/100 µg/ml; Gibco™ Life Technologies, Thermo Fisher Scientific), 1x Glutamax™ (Gibco™ Life Technologies). Cord blood (CB)-derived cells were isolated after parent written informed consent from cords/placentas of healthy full-term newborns (Department of Obstetrics, University Hospital Zurich and Triemli Hospital, Zurich). Processing of cells was performed by the biobank of the department of Medical Oncology and Hematology, University Hospital Zurich. CB-derived mononucleated cells were enriched by density gradient centrifugation (Ficoll-Paque Plus, GE healthcare, Chicago, IL, USA) and magnetically purified for CD34-positive cells by CD34 microbeads and LS columns (both Miltenyi Biotec). Subsequently, cells were cryo-preserved in 90% FCS (Gibco™ Life Technologies) + 10% DMSO (Sigma-Aldrich).

# CAR T-cell generation

Peripheral blood mononuclear cells (PBMCs) from healthy donors’ buffy coats (received after informed consent from the cantonal blood donation service, Zurich, Switzerland, and with ethical permission from the cantonal ethics committee Zurich) were enriched by density gradient centrifugation (Ficoll-Paque Plus, GE Healthcare). T-cells were purified by immune-magnetic methods using the “EasySep Human T cell isolation Kit” (Stemcell Technologies). Purified T-cells were cultured in a density of 1 – 2 x 10^6^ cells/ml T-cell medium (advanced RPMI 1640 media supplemented with 10% fetal bovine serum (FBS), penicillin/streptomycin (100 U/ml/100 µg/ml), 2 mM Glutamax, all Gibco™ Life Technologies)) with 100 U/ml recombinant human interleukin-2, IL-2 (Peprotech Thermo Fisher Scientific) and were activated with CD3/CD28 Dynabeads® (Thermo Fischer Scientific) in a 1:1 bead to cell ratio at d -1. On day 0, T-cell transduction was performed by adding concentrated lentiviral particles and hexamdimethrine bromide (Sigma-Aldrich) at a concentration of 8 µg/ml with consecutive centrifugation at 1500 x g for 1.5 hours at 32°C. Non-transduced T cells were used as control in all further experiments. Three days after activation, Dynabeads® were magnetically separated from the cells. Throughout the expansion phase, the T-cell medium was exchanged when considered necessary (mostly every other day). After an efficient expansion phase (> 100 x 10^6^ cells per condition, usually around d 8 after transduction), a magnetic purification for CD34-expression (CAR T-cell lentiviral vector, see 31) by CD34 microbeads and LS columns (both Miltenyi Biotec) was performed to achieve a >95% pure CAR T-cell population by Protein A-staining (Invitrogen, Carlsbad, CA, USA, see Supplementary Figure 8A). Non-transduced (control) T-cells and CAR T-cells were cryo-preserved after an additional expansion of maximal 12 days.

# Bright-field microscopy

At the day of analysis, cell suspensions of *in vitro* studies were bright-field photographed with a Leica DM5500-B microscope (Leica Microsystems, Wetzlar, Germany, using Leica LAS-X software) at 5x magnification (HC PL FLUOTAR 5x/0.15 Leica objective). Scaling bars as indicated in lower right corner.

# Flow cytometry-based analyses

Prior to staining, red blood cell lysis was performed on *in vivo* obtained single cells with ACK buffer (1.5 M NH_4_Cl, 100 mM KHCO_3_, 1 mM Na_2_EDTA, H_2_O) in a 1:40 dilution with an incubation time of 5 minutes at room temperature. Afterwards and in all *in vitro* experiments, cells were stained at the given timepoints first with Zombi Aqua™ (Biolegend, San Diego, CA, USA) to distinguish between live and dead cells. Second, cells were stained with membrane bound dye (APC / CellVue® Claret Far Red Fluorescent Cell Linker Mini Kit, Sigma-Aldrich) at the manufacturer’s instructions for cell proliferation determination and / or the given fluorochrome-coupled antibodies (Supplementary Table 1). Acquisition of data was performed using a „BD LSRFortessa™ II flow cytometer“, and the software „BD FACSDiva™“ (all Becton Dickinson). Analysis was done in “FlowJo” (v10.0.7, TreeStar Inc., Becton Dickinson). Specific lysis of target cells has been calculated using the ratio of alive CD117+ cells in co-culture with-CAR T-cells to alive CD117+ cells in control condition:

$$specific lysis \left[ \% \right]=\frac{(1-number of alive target cells in CAR T cell coculture)}{(number of alive target cells in ctrl coculture)}*100$$

# Cytokine analysis by ELISA

On the day of analysis, cell suspension co-cultures were spun down and levels of secreted IL-2 in the supernatant were quantified by enzyme-linked immunosorbent assay (ELISA) according to manufacturer’s instructions (ELISA MAX Deluxe Set, Biolegend). Absorbance of color cover was measured at 450 nm was read out on Mini ELISA Plate Reader™ (Biolegend).

# *In vivo* studies

Animals and animal experiments were kept and performed in accordance with the approved protocols and animal license (135/2021) by Cantonal Veterinary Office Zurich. Humanized mice (MITRG-SKI and MITRG-SKI-6) were generated and bred in-house as previously described^9–11^. NSG-mice were either in-house bred or purchased from Charles River (Germany) (6-8 weeks old). There was no sex preference of the experimental animals. Detailed information on each *in vivo* experiment is given in the-cartoons related to each *in vivo* experiment figure.

For the *in vivo* studies, ROSA^KIT D816V^ were lentivirally transduced to express GFP and Luciferase (pCDH-EF1α-Luciferase-T2A-GFP (System Biosciences, Palo Alto, CA, USA)) for better trackability of human cells in mice. For this purpose, to 1 x 10^6^ ROSA^KIT D816V^ cell concentrated lentiviral particles and hexamdimethrine bromide (Sigma-Aldrich) at a concentration of 8 µg/ml were added. Consecutively, cells were centrifuged at 1500 x g for 1.5 hours at 32°C. After an expansion phase, cells were subjected to a limited dilution, and the clone with high expression of GFP in flow cytometry analysis was picked for further purposes.

## ROSA^KIT D816V+GFP/Luciferase^, *KIT* D816V+ iPS-derived cell and CD45+/CD117+ sorted patient BM cell transplantation into mice

Mice (8-12 weeks old) were sublethally irradiated with 100 cGy (RS-2000 irradiator, Rad Source). 4-6 hours after irradiation human cells were intravenously (i.v.) transplanted. In order to track human cell engraftment, blood withdrawal and subsequent analysis by flow cytometry or *in vivo* bioluminescence imaging was performed (see dedicated section).

## *In vivo* bioluminescence imaging

Mice were anesthetized with Isoflurane and i.p.-injected with 150 mg/kg body weight IVISbrite™ D-Luciferin (diluted at 15 g/l in PBS, PerkinElmer, Waltham, MA, USA). After an incubation time of six minutes, bioluminescence was measured using an “IVIS® Lumina X5 in vivo imaging system” and analyzed with the software “Living Image” (both by PerkinElmer). Total Flux of the signal [p/s] was recorded from the “region of interest” (ROI) of the given mouse body. Luminescence scale is given for the individually analyzed group.

## Terminal analysis

Upon termination of the experiment and euthanasia of the experimental animals, the organs of interest (e.g. femora, tibiae, liver, spleen) were harvested and further processed. In brief, for histological analysis organs were immediately transferred to 4% buffered paraformaldehyde (PFA, Santa Cruz Biotechnology, Dallas, TX, USA). For flow cytometry analysis, long-bones were flushed with FACS buffer (PBS (Gibco™ Life Technologies), 2% FBS (Gibco™ Life Technologies), 2 mM EDTA (Thermo Fisher Scientific)), organs were crushed with a blunt plastic tool and subsequently strained (40 µm cell strainer) to get a single cell solution.

## Transplantation of primary human CB cells into mice and analysis of MC distribution

On the day of experiment initiation, CB cells were thawed and intravenously transplanted in beforehand irradiated NSG, MITRG-SKI and MITRG-SKI-6 mice. Every second week, sublingual blood was withdrawn to track human engraftment. As soon as an animal of the dedicated mouse strain group met termination criteria (defined by anemia-related symptoms like staggered breathing due to mouse hematopoiesis replacement by human cells), all animals of that group were euthanized and terminally analyzed by investigating human (MC) engraftment in PB, BM, spine, spleen, liver, skin, lung and intestine.

## *In vivo* engraftment of patient-derived iPS cells

As described in 1.1, on day 6 after harvesting HSPCs from spinEBs irradiated mice of all strains (NSG, MITRG-SKI, MITRG-SKI-6) were transplanted with >60% CD34+ HSPCs intravenously (in total 0.28 x 10^6^ cells per mouse CD34+ HSPCs). Sublingual peripheral blood was withdrawn to determine human cell engraftment. After more than 180 days the experiment was aborted and terminal analysis of PB, BM and the spine was performed.

## *In vivo* engraftment of SM patient sample

As described in 1.2, patients samples with a confirmed diagnosis of SM were thawed and subsequently subjected to fluorescent activated cell sorting (BD FACSAria cell sorter, Becton Dickinson) to obtain a hCD117+/hCD45+ (Supplementary Table 1) population which was intravenously injected in mice of all strains (NSG, MITRG-SKI, MITRG-SKI-6). As soon as the mice met termination criteria (see above), the animals were euthanized, and terminal analysis of PB, BM, spleen and liver was performed.

## CAR T-cell efficacy in human cell line engrafted mouse models

5 x 10^6^ ROSA^KIT D816V +GFP/Luciferase^ cells (or PBS control) were transplanted in 8-12 weeks old NSG mice of both genders as described in 6.1. *In vivo* bioluminescence imaging (as described in 6.2) were grouped according to the emitted flux signal (PBS injection, control T-cells,-CAR T-cells) was performed on day 7 after transplantation and throughout the experiment. After grouping, PBS, 10 x 10^6^-CAR T-cells or control T-cells were i.v. injected on day 7 and day 21 after cell line transplantation. Blood withdrawal for determination of human engraftment and human CD3 positive cells presence by flow cytometry analysis was performed at the given timepoints as described in 4. At the study endpoint, terminal analysis of peripheral blood (PB), bone marrow (BM), spleen and liver was performed as described in 6.3.

## Histology and Immunohistochemistry

Histology samples were analyzed according to clinical protocols at the department of pathology at the University Hospital Zurich. Given mouse tissue (femura, tibiae, spleen, liver) were fixed in 4% buffered paraformaldehyde (PFA, Santa Cruz Biotechnology). Bones were decalcificated in EDTA with MoL-DECALCIFIER on a DecalMATE instrument (Milestone Medical, Valbrembo, Italy). Tissues were handled on a Leica PELORIS 3 tissue processor (Leica Biosystems) followed by paraffin embedding. After tissue section (2-3 µm) and mounting on SuperFrost Plus slides (Thermofisher Scientific) slides were stained with hematoxylin and eosin (HE) and / or subjected to immunohistochemical (IHC) staining. For IHC the anti-hCD117 antibody (clone YR145, rabbit monoclonal, Cell Marque, Rocklin, CA, USA) at an 1:200 dilution, after 32 minutes of incubation with an OptiView DAB detection kit (Ventana Medical Systems, Oro Valley, AZ, USA) on a Ventana Benchmark ULTRA system (Ventana Medical Systems) was used. Samples were analyzed by light microscopy, digitalization was done with a Nano Zoomer C9600 scanner (Hamamatsu Photonics, Hamamatsu, Japan).

**Supplementary tables**

Supplementary Table 1. Used antibodies for this study (h: anti-human, m: anti-murine).

| **Surface staining** | **Fluorochrom** | **Clone** | **Manufacturer/**  **Catalog number** | **originating species** | **Dilution** |
| --- | --- | --- | --- | --- | --- |
| **hCD3** | BV711 | UCHT1 | Biolegend  300463 | mouse | 1:200  (1:100 *in vivo*) |
| **hCD33** | APC | P67.6 | Biolegend  366605 | mouse | 1:100 |
| **hCD33** | PerCP Cy5.5 | P67.6 | Biolegend  366615 | mouse | 1:200 |
| **hCD34** | FITC | QBEND-10 | Invitrogen  # MA1-10204 | mouse | 1:20 |
| **hCD34** | PE | QBEND-10 | Invitrogen  # MA1-10205 | mouse | 1:20  (1:10 *in vivo*) |
| **hCD45** | eFluor™ 450 | HI30 | Invitrogen  # 48-0459-42 | mouse | 1:200 |
| **hCD45** | APC-Cy7-A | 5B1 | Miltenyi  130-113-115 | mouse | 1:200  (1:100 *in vivo*) |
| **mCD45** | PerCP Cy5.5 | 30-F11 | Biolegend  103131 | rat | 1:100 |
| **hCD69** | BV605 | FN50 | Biolegend  310937 | mouse | 1:200 |
| **hCD117** | PeCy7 | 104D2 | Invitrogen  # 25-1178-42 | mouse | 1:200  (1:100  *in vivo* and for sorting) |
| **hCD123** | Brilliant Violet 421™ | 6H6 | Biolegend  306017 | mouse | 1:200 |
| **hCD371** | APC | 50C1 | Biolegend  353605 | mouse | 1:200 |
| **Protein A– FITC Conjugate** | FITC | n/a | Invitrogen  # 200-102-077 | n/a | 1:20 |
| **live / dead** | Zombi Aqua™ | n/a | Biolegend  423101 | n/a | 1:800 |
| **membrane dye / proliferation marker** | APC / CellVue® Claret Far Red Fluorescent Cell Linker Mini Kit for General Membrane Labeling | n/a | Sigma-Aldrich  12352207 | n/a | as manufacturer‘s instructions |
|  | | | | | |
| **patient sample characterization** | | | | | |
| **hCD2** | PC7 | 39C1.5 | Beckman Coulter  737662 | rat | 1:8 |
| **hCD25** | ECD | B1.49.9 | Beckman Coulter  6607112 | mouse | 1:12 |
| **hCD33** | PE | D3HL60.251 | Beckman Coulter  A07716 | mouse | 1:4 |
| **hCD45** | APC | J33 | Beckman Coulter  IM2473 | mouse | 1:8 |
| **hCD117** | PC5 | 104D2D1 | Beckman Coulter  IM2733 | mouse | 1:12 |

Supplementary Table 2. Patient sample characteristics.

|  | **diagnosis** | **BM infiltration (smear)** | ***KIT*^D816V^  allele burden** | **additional mutations** | **expression aberrant markers** | **serum tryptase, µg/l** | **chromosomal analysis** |
| --- | --- | --- | --- | --- | --- | --- | --- |
| **Patient 1**  male  73 years | aleukemic chronic MCL (first diagnosed 2017) | 20% | 45% | MYD88 | CD2, CD25 | 350 | 45,X,-Y [11]  47,XY,+8 [1]  46,XY [10] |
| **Patient 2**  male  60 years | aleukemic chronic MCL (first diagnosed 2016) | 30-35% | 2,3% | - | CD2, (CD25) | 76,4 | - |

Supplementary Table 3. Composition of used cell line media.

| **Cell line** | **Ingredient** | **Manufacturer** | **Concentration** |
| --- | --- | --- | --- |
|  |  | | |
| **HMC-1.1^KIT V560G^, HMC-1.2^KIT V560G, D816V^, ROSA^KIT D816V^** | IMDM + L-glutamine | Gibco™ |  |
|  | FCS | Gibco™ | 10% |
|  | Pen/Strep | Gibco™ | 100 µg/U /ml |
|  |  | | |
| **ROSA^KIT WT^** | IMDM + L-glutamine | Gibco™ |  |
|  | FCS | Gibco™ | 10% |
|  | Pen/Strep | Gibco™ | 100 µg/U /ml |
|  | SCF | Miltenyi Biotec | 80 ng/ml |
|  |  | | |
| **MCPV-1** | IMDM + L-glutamine | Gibco™ |  |
|  | FCS | Gibco™ | 10% |
|  | Pen/Strep | Gibco™ | 100 µg/U /ml |
|  | SCF | Miltenyi Biotec | 100 ng/ml |
|  |  | | |
| **LAD** | StemPro™ | Gibco™ |  |
|  | L-glutamine | Gibco™ | 2 mM |
|  | Pen/Strep | Gibco™ | 100 µg/U /ml |
|  | SCF | Miltenyi Biotec | 100 ng/ml |

Supplementary Table 4. Media composition for iPS cell differentiation to mature MCs.

| **Ingredient** | **Manufacturer** | **Concentration** | **spinEBs** | | | **HSPCs** | **MC maturation** |
| --- | --- | --- | --- | --- | --- | --- | --- |
|  |  |  | day 0-2 | day 2-7 | day 8-14 | day 0-20 | day 0-70 |
| basal medium | | | | | | | |
| IMDM | Gibco™ | 48% |  |  |  |  |  |
| HAM’s F-12 | ThermoScientific | 48% |  |  |  |  |  |
| chem.def.  lipid concentrate | Gibco™ | 1% |  |  |  |  |  |
| GlutaMAX™ supplement | Gibco™ | 2 mM |  |  |  |  |  |
| 1-thioglycerol (MTG) | Sigma-Aldrich | 400 µM |  |  |  |  |  |
| BSA | Sigma | 0.5% |  |  |  |  |  |
| human albumin | CSL Behring | 0.5% |  |  |  |  |  |
| L-ascorbic acid | Stemcell Technologies | 50 µg/ml |  |  |  |  |  |
| supplements | | | | | | | |
| human  holo-transferrin | Sigma-Aldrich | 6 µg/ml |  |  |  |  |  |
| recombinant human FGF | Peprotech | 10 ng/ml |  |  |  |  |  |
| Rho kinase inhibitor | abcam | 10 µM/l |  |  |  |  |  |
| human BMP-4 | Miltenyi Biotec | 10 ng/ml |  |  |  |  |  |
| human SCF | Miltenyi Biotec | 50 ng/ml |  |  |  |  |  |
| recombinant human VEGF | Peprotech | 10 ng/ml |  |  |  |  |  |
| Pen/Strep | Gibco™ | 50 µg/U ml |  |  |  |  |  |
| human IL-3 | Miltenyi Biotec | 30 ng/ml |  |  |  |  |  |
| recombinant human FLT3-ligand | Peprotech | 50 ng/ml |  |  |  |  |  |
| recombinant human IL-6/IL-6R | R&D systems | 25 ng/ml |  |  |  |  |  |

Supplementary Table 5. Overview of all *in vivo* experiments.

**Supplementary figures**

Supplementary Figure 1

**B**


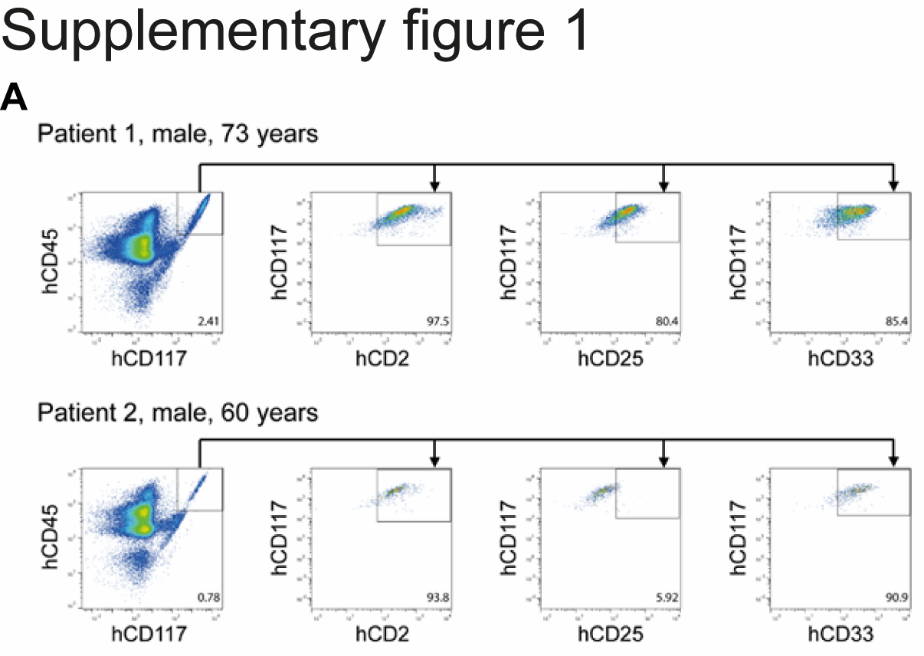


**A**


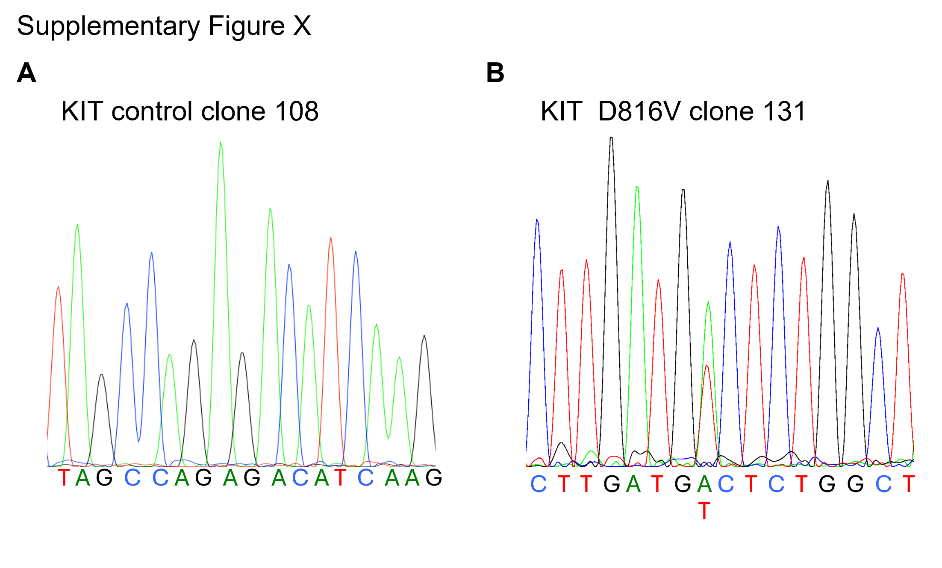


**Supplementary Figure 1:** **Genotype of iPS cell clones analyzed by Sanger sequencing and patient sample characterization by flow cytometry.** (A) iPS cell-derived progenitor cells without *KIT* mutation (KIT control clone 108) and iPS cell-derived progenitor cells with *KIT* D816V mutation (A) to Thymine (T) (KIT D816V clone 131). (B) Treatment-related bone marrow biopsy was performed on the given two patients with a confirmed diagnosis of SM (in these cases chronic aleukemic MCL, Supplementary Table 2). Used antibodies for staining are given in Supplementary Table 1.


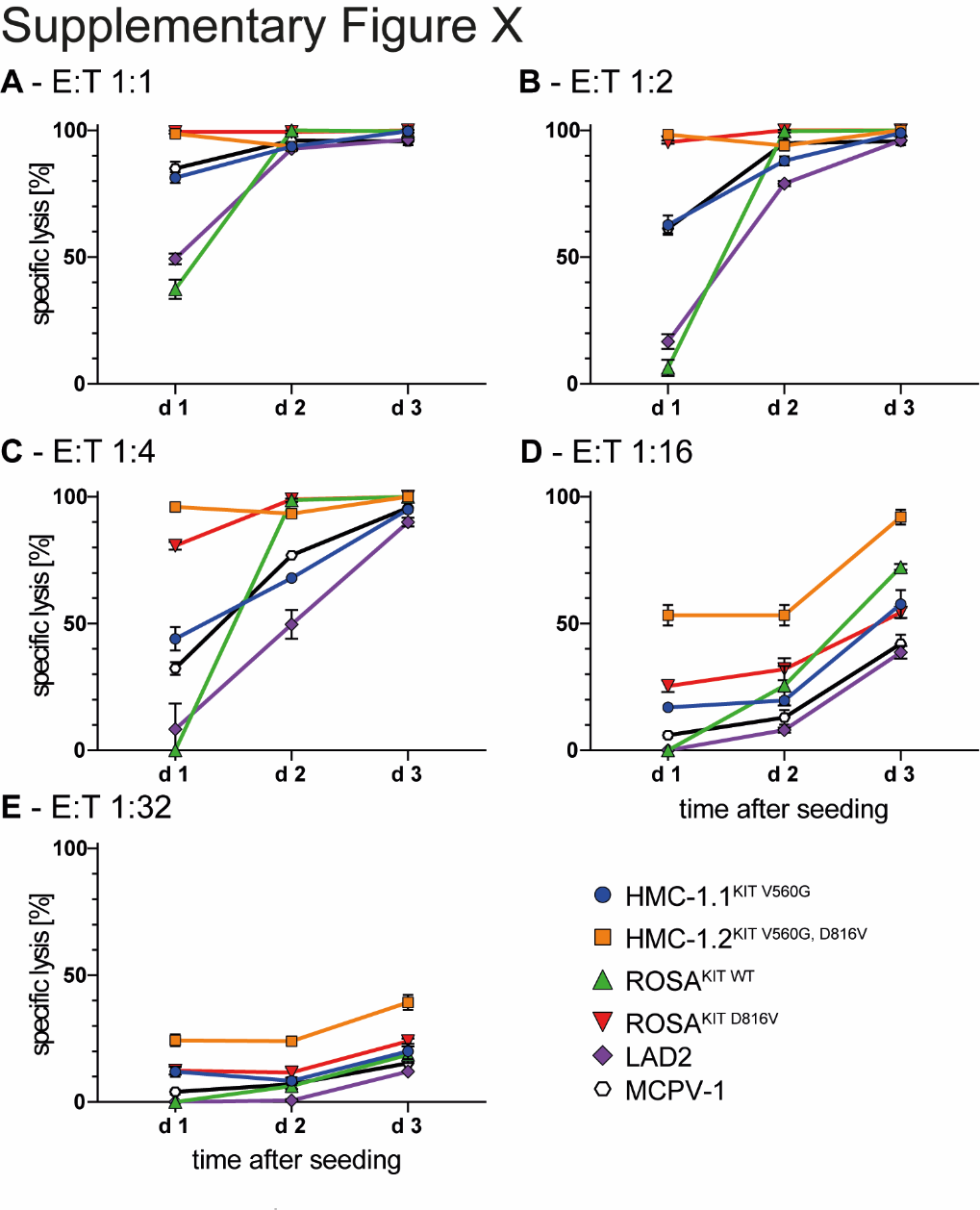
Supplementary Figure 2

**Supplementary Figure 2.** **Anti-human CD117-CAR T-cells efficiently lyse human MC lines in various effector to target (E:T) ratios, regardless of their *KIT* D816V mutation status.** (A-E) Specific cell lysis of various human MC lines (with or without *KIT* D816V mutation, as indicated) after one, two, or three days of co-culturing with anti-human CD117-CAR T-cells. Lysis percentages were calculated relative to control conditions using untransduced (control: ctrl.) T-cells). Effector-to-target (E:T) ratios ranging from 1:1 to 1:32 are depicted (mean ± SD, blue circle: HMC-1.1^KIT V560G^, orange square: HMC-1.1^KIT V560G, D816V^, green triangle up: ROSA^KIT WT^, red triangle down: ROSA^KIT D816V^, purple diamond: LAD2, white hexagon: MCPV-1). Data shows one example of three independently performed experiments with three healthy donor-derived CAR T-cells, each plated in triplicate wells.


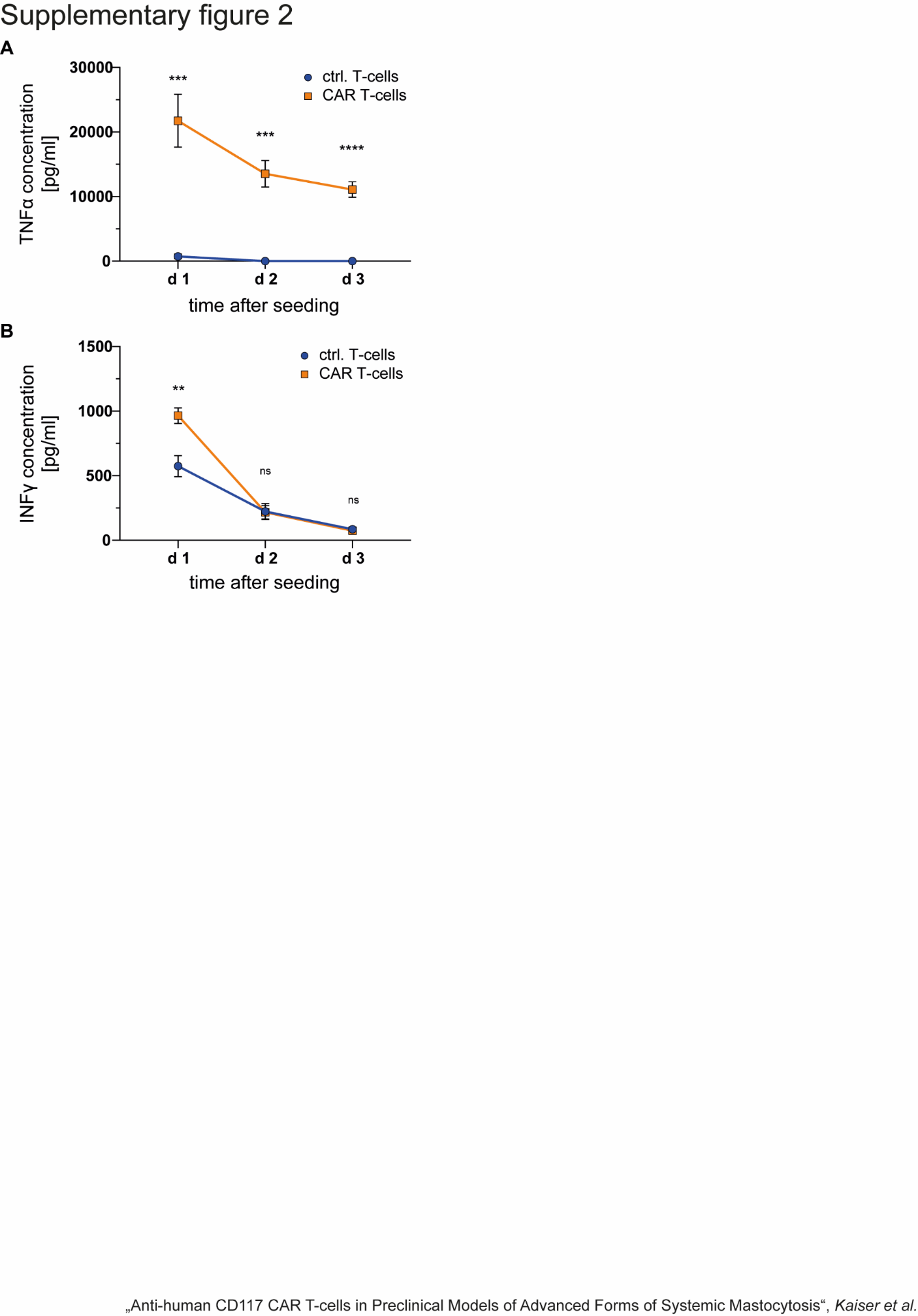
Supplementary Figure 3

**Supplementary Figure 3: Production of proinflammatory cytokines by CAR T-cells upon co-culture with target cells.** (A) TNFα and (B) INFγ production in co-culture supernatant (ctrl. T-cells or CAR-T-cells as indicated with ROSA^KIT D816V^ cell line) measured by ELISA (mean ± SD, E:T ratio of 1:1). Statistics: unpaired t-test: ns; * p <0.05; ** p <0.01; *** p <0.001; **** p <0.0001. Data shows one example of three independently performed experiments with three healthy donor-derived CAR T-cells, each plated in triplicate wells.


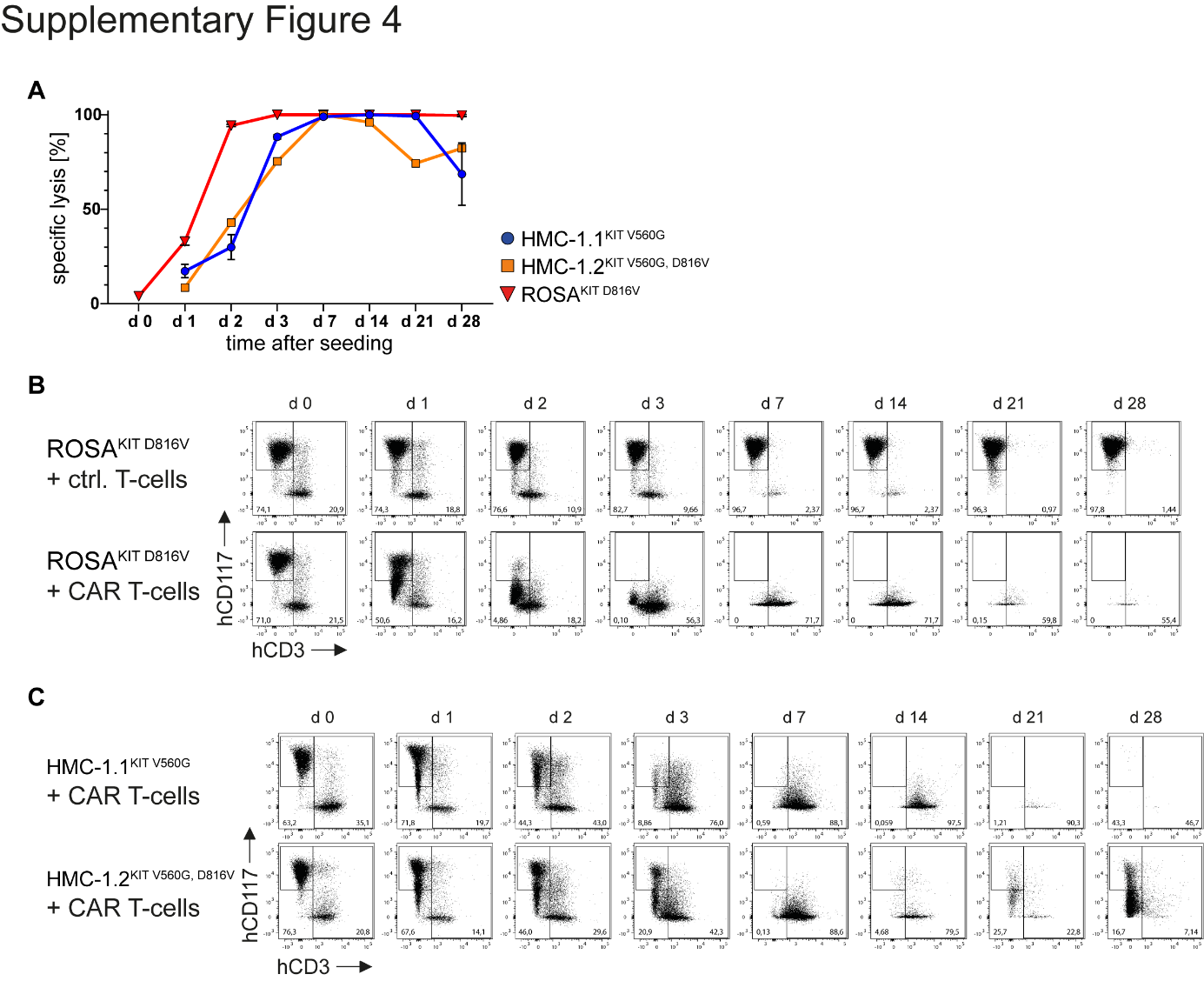
Supplementary Figure 4

**Supplementary Figure 4: Anti-CD117-CAR T-cells control human MC lines with or without the *KIT* D816V mutation in long-term cultures in an E:T 1:4 effector to target ratio.** (A) Specific cell lysis of human MC lines with or without *KIT* D816V mutation, as indicated, over a 28-day period of co-culture with anti-human CD117-CAR T-cells. Lysis in percent was calculated relative to control using untransduced T-cells at an E:T ratio of 1:4 (mean ± SD). (B) Representative flow cytometry plots of ROSA^KIT D816V^ human MC line (hCD117+ cells) alongside T-cells (hCD3+ cells: either ctrl. T-cells or CAR T-cells) throughout the observation period of co-culture (day 0 to day 28). (C) Representative flow cytometry plots of HMC^KIT V560G^ and HMC^KIT V560G, D816V^ human MC lines (hCD117+ cells) alongside CAR T-cells (hCD3+ cells) throughout the observation period of co-culture as in (B).


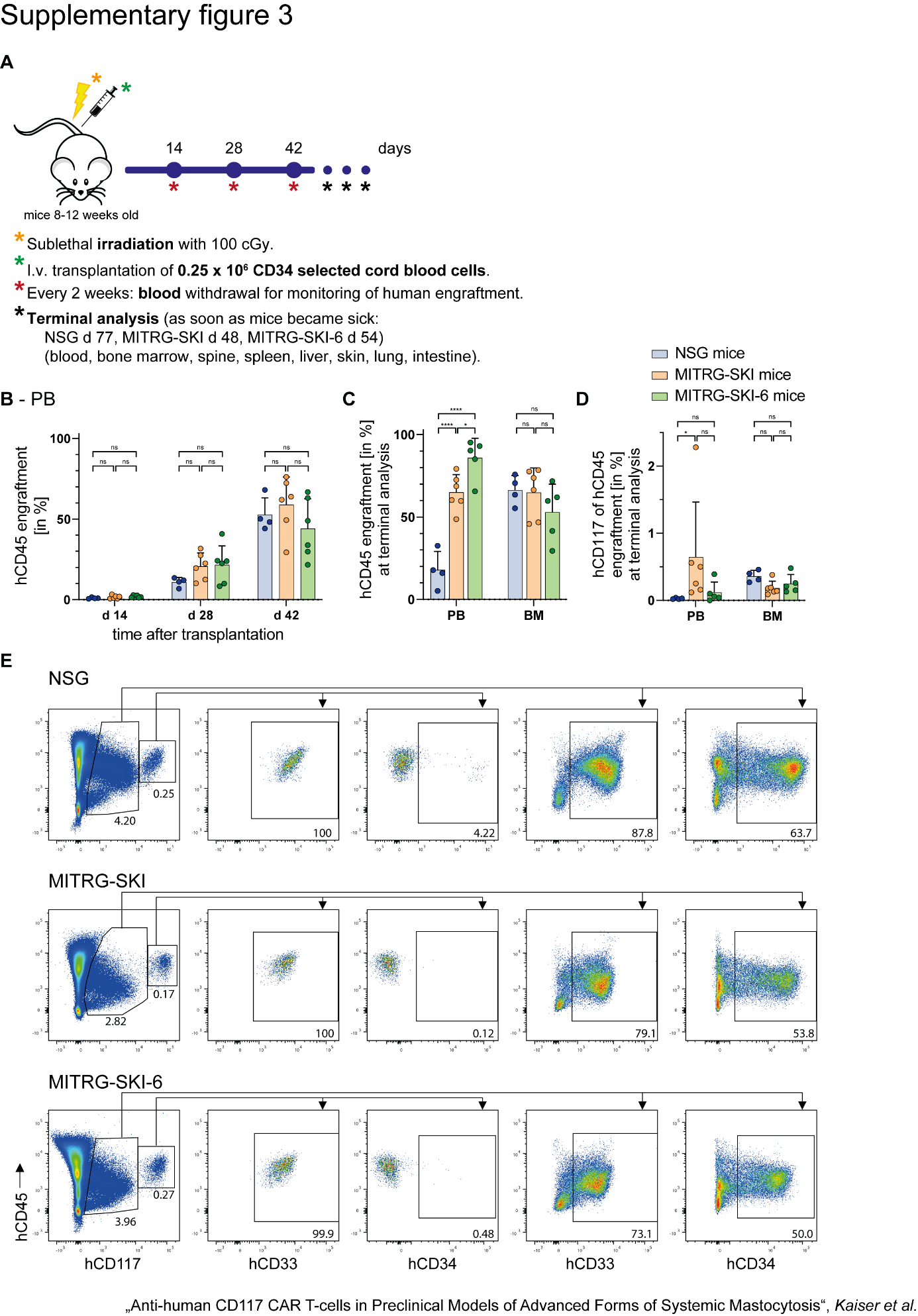
Supplementary Figure 5

**Supplementary Figure 5:** **Comparison of *in vivo* development and engraftment of cord blood-derived human MCs in NSG, MITRG-SKI, and MITRG-SKI-6 mice.** (A) Schematic experimental setup: 0.25 x 10^6^ CD34-selected cord blood cells were intravenously (i.v.) transplanted into sublethally irradiated 8-12-week-old NSG (n = 4), MITRG-SKI (n = 6), or MITRG-SKI-6 (n = 6) mice. Peripheral blood (PB) was withdrawn every two weeks to assess human engraftment by flow cytometry. When mice of a given strain reached experimental endpoints, all mice in that group were sacrificed for terminally analysis. One mouse of the MITRG-SKI-6 group was found dead for unknown reasons two days before terminal analysis. (B) Monitoring of human engraftment (% hCD45+ cells) in PB at two-week intervals post-transplantation (mean ± SD). (C) Flow cytometric analysis of human engraftment (hCD45+) in PB and bone marrow (BM) (mean ± SD). (D) Flow cytometric analysis of MC percentage (hCD117+) of hCD45+ in PB and BM (mean ± SD). (E) Representative scatter plots and characterization of hCD117+/hCD45^int^ and hCD117^int^/CD45^int^ cell population in BM. Each plot corresponds to an individual representative mouse of the given strain. For all statistics: Brown–Forsythe and Welch one-way ANOVA followed by Dunnett T3 post-hoc tests; for non-normally distributed data: Mann–Whitney test and Kruskal–Wallis test with Dunn’s multiple comparisons: ns; * p <0.05; ** p <0.01; *** p <0.001; **** p <0.0001.
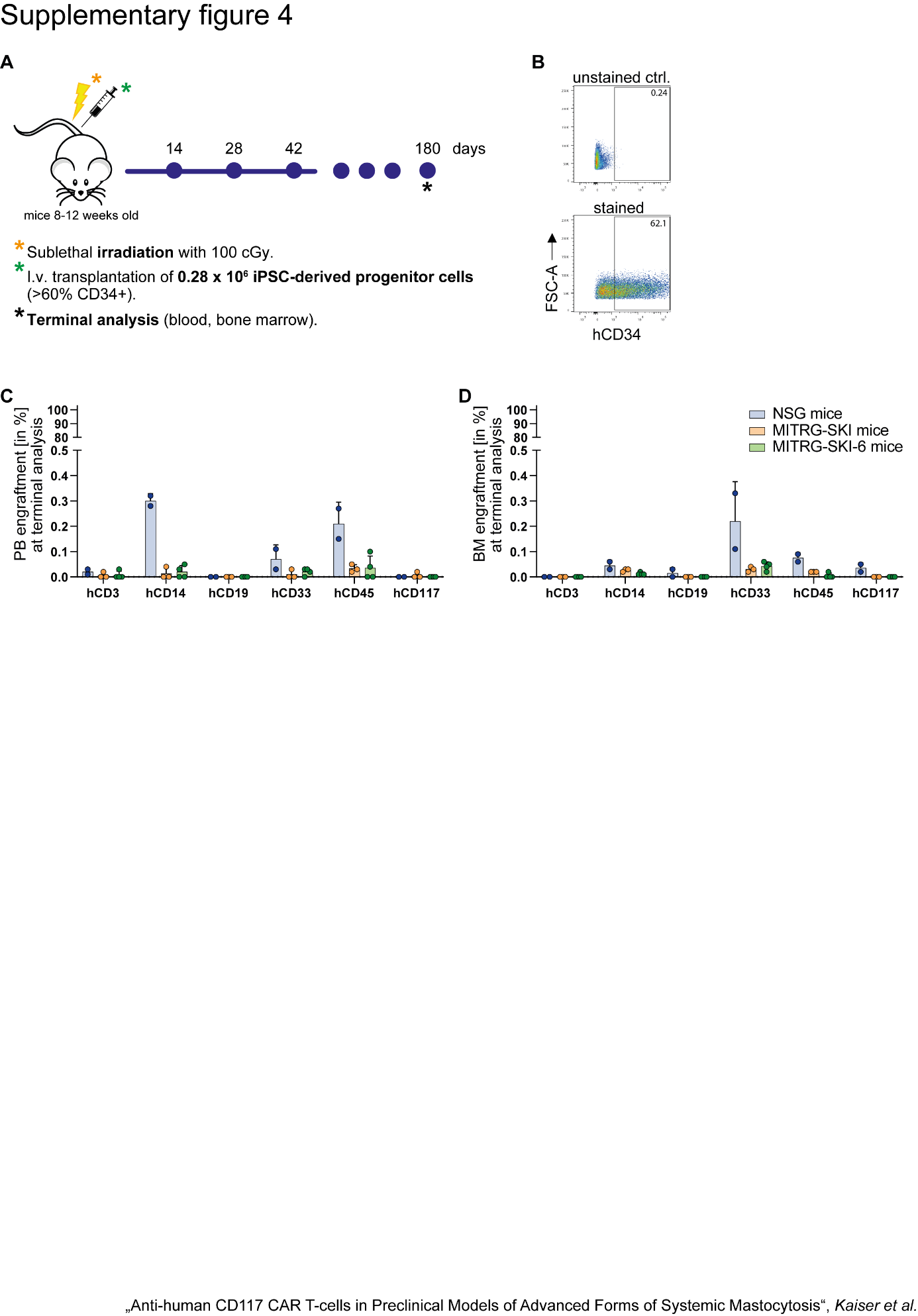
Supplementary Figure 6

**Supplementary Figure 6:** **Comparison of *in vivo* engraftment of iPS cell-derived progenitor cells in NSG, MITRG-SKI, and MITRG-SKI-6 mice.** (A) Schematic experimental setup: 0.28 x 10^6^ iPS cell-derived progenitor cells (>60% CD34+) were intravenously (i.v.) transplanted into sublethally irradiated 8-12-week-old NSG ( n = 3), MITRG-SKI (n = 3), or MITRG-SKI-6 mice (n = 3). After more than 180 days of experimental follow-up all mice were sacrificed and terminally analyzed. One mouse of the NSG group died in anesthesia. (B) Dot plot diagram displaying CD34 expression of iPS cell-derived progenitor cells on the day of i.v. injection into given mouse strains. Characterization of engrafted human cells in PB (C) and BM (D) at terminal analysis (mean ± SD).


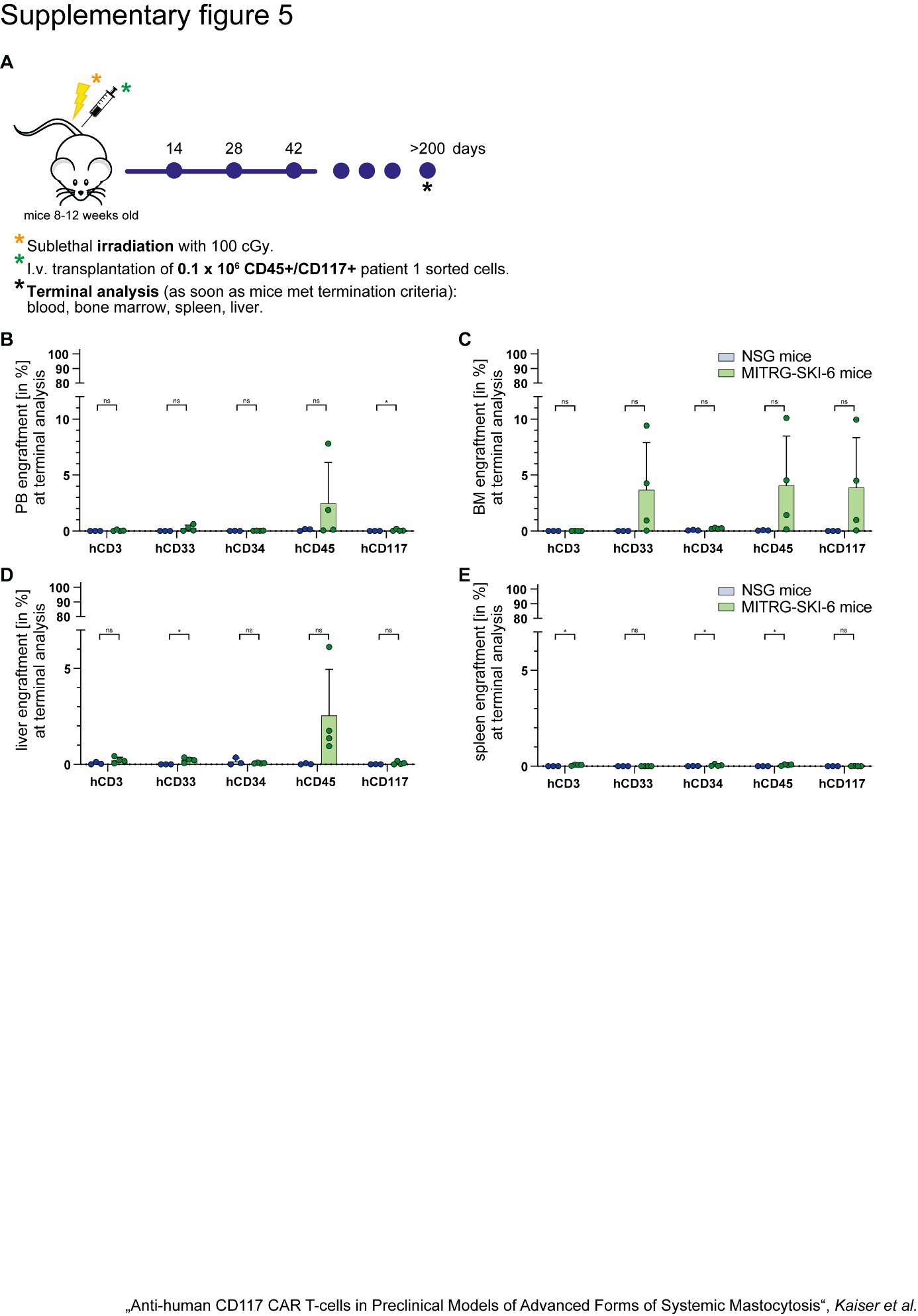
Supplementary Figure 7

**Supplementary Figure 7:** **Comparison of *in vivo* engraftment of SM patient sample in NSG and MITRG-SKI-6 mice.** (A) Schematic experimental setup: 0.1 x 10^6^ hCD117+/hCD45+ sorted MCL patient sample cells (patient 1, see Supplementary Table 2) were intravenously (i.v.) transplanted into sublethally irradiated 8-12-week-old NSG (n = 4) or MITRG-SKI-6 mice (n = 5). As soon as mice met the termination criteria all mice were sacrificed and terminally analyzed. In the experiment one mouse of each group was found dead for unknown reasons. Characterization of engrafted human cells in PB (B), BM (C), liver (D) and spleen (E) at terminal analysis (mean ± SD). For all statistics: unpaired t-test: ns; * p <0.05; ** p <0.01; *** p <0.001; **** p <0.0001.


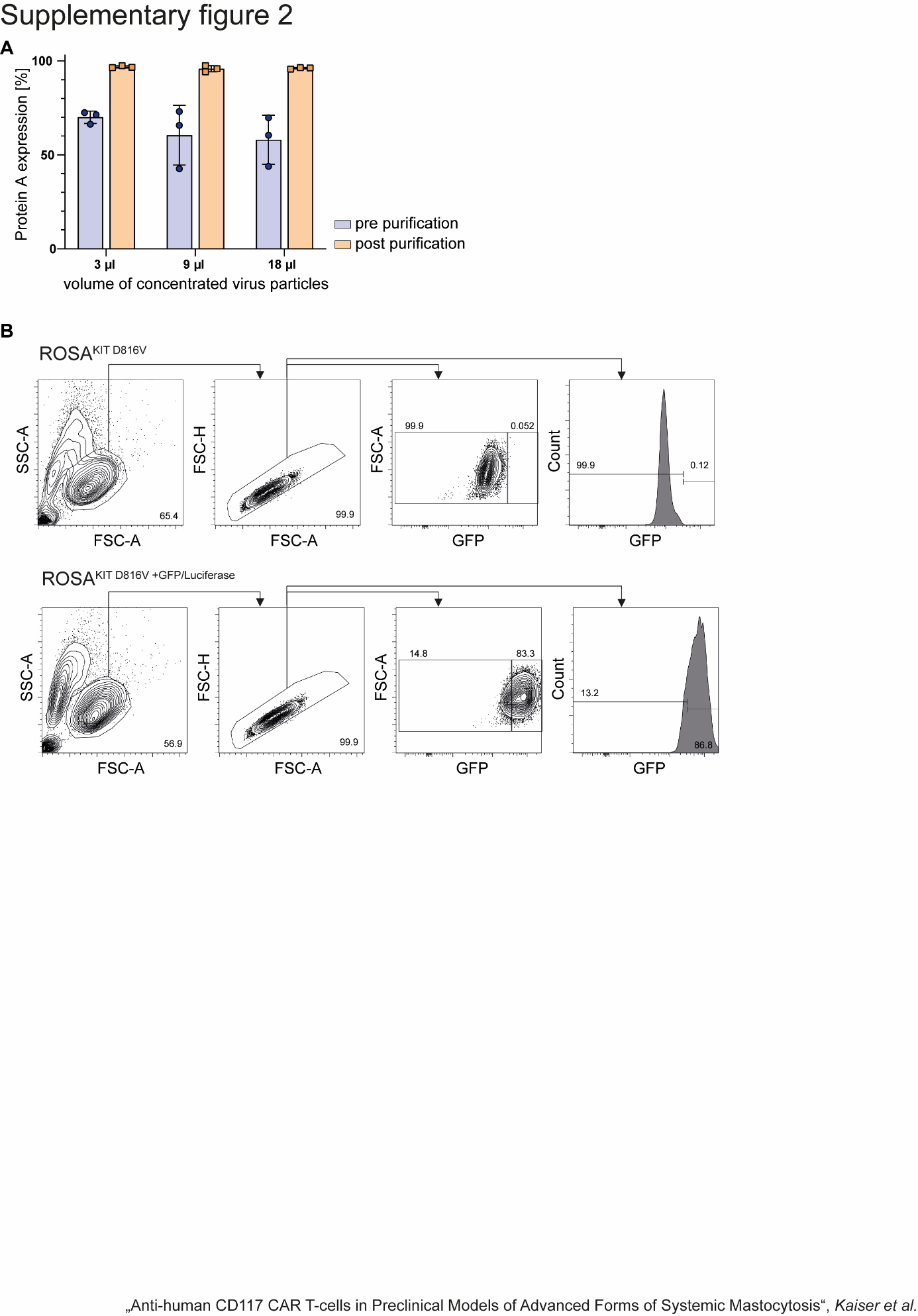
Supplementary Figure 8

**Supplementary Figure 8: CAR T-cell purification and ROSA^KIT D816V^ GFP/Luciferase-transduction.** (A) T-cell expression of Protein A (CAR) pre and post purification after transduction with the given volumes of concentrated virus particles (mean ± SD). (B) GFP expression of ROSA^KIT D816V^ pre and post lentiviral GFP/Luciferase-transduction measured by flow cytometry.


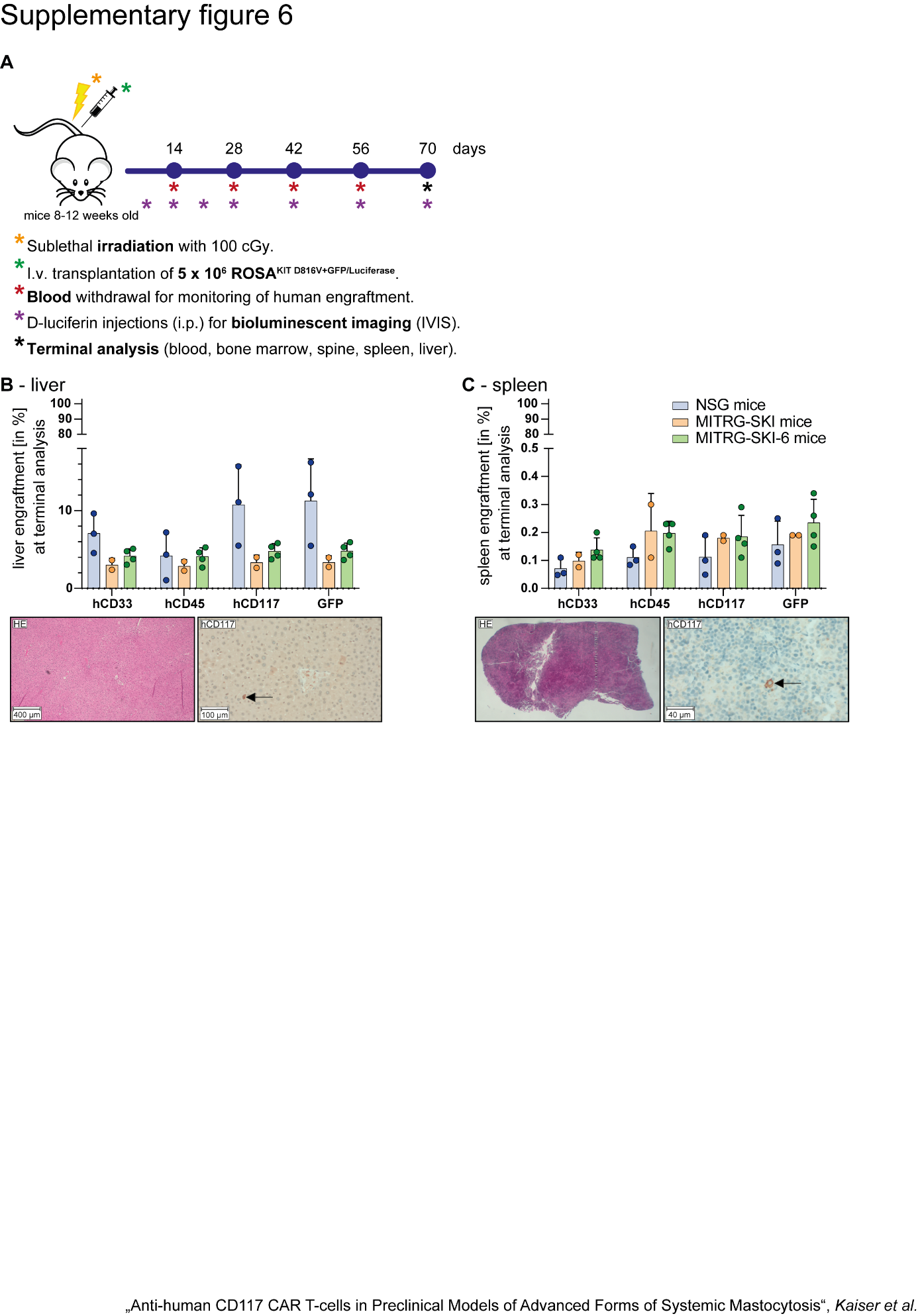
Supplementary Figure 9

**Supplementary Figure 9:** **Comparison of *in vivo* engraftment of ROSA^KIT D816V +GFP/Luciferase^ human MC line in NSG-, MITRG-SKI- and MITRG-SKI-6 mice.** (A) Schematic experimental setup: 5 x 10^6^ ROSA^KIT D816V +GFP/Luciferase^ cells were intravenously (i.v.) transplanted into sublethally irradiated 8-12-week-old NSG (n = 4), MITRG-SKI (n = 3), or MITRG-SKI-6 (n = 4) mice. Peripheral blood (PB) was withdrawn every two weeks to assess human engraftment by flow cytometry. As indicated, cell line engraftment was checked by *in vivo*bioluminescence signal. Mouse 1 of the NSG and mouse 3 of MITRG-SKI group died in anesthesia for engraftment unrelated reasons. After 70 days of transplantation, all mice were analyzed. Characterization of engrafted human cells in liver (B) and spleen (C) at terminal analysis (mean ± SD). Representative HE-stained sections of corresponding organs of MITRG-SKI-6 mice. Additional immunohistochemical (IHC) photomicrographs for MCs (hCD117) indicated by an arrow (scaling bars as indicated in lower left corner).


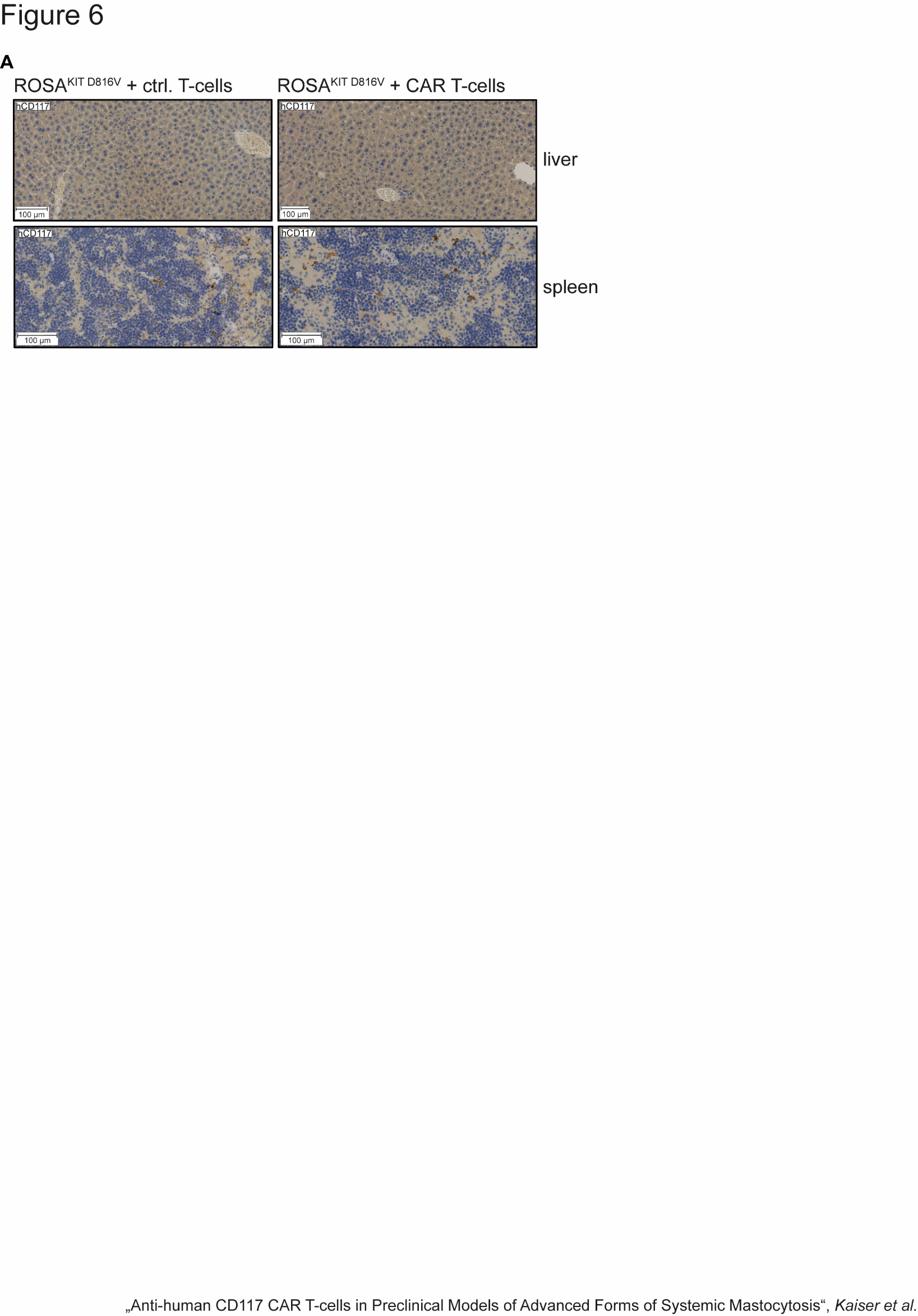
Supplementary Figure 10

**Supplementary Figure 10.** **Human** **CD117+ cells in liver and spleen of ROSA^KIT D816V +GFP/Luciferase^ MC engrafted NSG mice after untransduced T-cells and CAR T-cell treatment.** (A) Representative anti-human CD117 IHC analysis for MCs in liver and spleen of mice treated with untransduced T-cells (ctrl.T-cells, left) and anti-human CD117-CAR T-cells (right) (scaling bars, indicate 100 µm).

# Supplementary references

1 Hoermann G, Blatt K, Greiner G, Putz EM, Berger A, Herrmann H *et al.* CD52 is a molecular target in advanced systemic mastocytosis. *The FASEB Journal • Research Communication* 2014. doi:10.1096/fj.14-250894.

2 Kirshenbaum AS, Yin Y, Bruce Sundstrom J, Bandara G, Metcalfe DD. Description and characterization of a novel human mast cell line for scientiﬁc study. *Int J Mol Sci* 2019; **20**. doi:10.3390/ijms20225520.

3 Saleh R, Wedeh G, Herrmann H, Bibi S, Cerny-Reiterer S, Sadovnik I *et al.* A new human mast cell line expressing a functional IgE receptor converts to tumorigenic growth by KIT D816V transfection. *Blood* 2014; **124**. doi:10.1182/blood-2013-10.

4 Butterfield JH, Weiler D, Dewald G, Gleich GJ. Establishment of an immature mast cell line from a patient with mast cell leukemia. *Leuk Res* 1988; **12**: 345–355.

5 Atakhanov S, Christen D, Rolles B, Schüler HM, Panse J, Chatain N *et al.* Towards personalized medicine with iPS cell technology: a case report of advanced systemic mastocytosis with associated eosinophilia. Ann. Hematol. 2022; **101**: 2533–2536.

6 Toledo AMAS, Gatz M, Sontag S, Karoline V, Rossetti G, Sechi AS *et al.* Nintedanib Targets KIT D816V Neoplastic Cells Derived from Induced Pluripotent Stem cells of Systemic Mastocytosis. *Blood* 2021; **137**: 2070–2084.

7 de Toledo MAS, Fu X, Maié T, Buhl EM, Götz K, Schmitz S *et al.* KIT D816V Mast Cells Derived from Induced Pluripotent Stem Cells Recapitulate Systemic Mastocytosis Transcriptional Profile. *Int J Mol Sci* 2023; **24**: 5275.

8 Sontag S, Förster M, Qin J, Wanek P, Mitzka S, Schüler HM *et al.* Pluripotent Stem Cells Modelling IRF8 Deficient Human Hematopoiesis and Dendritic Cell Development with Engineered. *Stem Cells* 2017; **35**: 898–908.

9 Rongvaux A, Willinger T, Martinek J, Strowig T, Gearty S V., Teichmann LL *et al.* Development and function of human innate immune cells in a humanized mouse model. *Nat Biotechnol* 2014; **32**: 364–372.

10 Ellegast JM, Rauch PJ, Kovtonyuk L V., Müller R, Wagner U, Saito Y *et al.* inv(16) and NPM1mut AMLs engraft human cytokine knock-in mice. *Blood* 2016; **128**: 2130–2134.

11 Theocharides APAA, Rongvaux A, Fritsch K, Flavell RA, Manz MG. Humanized hemato-lymphoid system mice. *Haematologica* 2016; **101**: 5–19.
